# Cellular signaling within aged skeletal muscle reveals a dysregulated stress‐induced remodeling response following volumetric muscle loss in female mice

**DOI:** 10.14814/phy2.71022

**Published:** 2026-07-23

**Authors:** Krista M. Habing, Tyler J. Sagendorf, Cynthia A. Alcazar‐Daleo, James A. Sanford, Damon Leach, Joshua C. Vanderpool, Chelsea M. Hutchinson‐Bunch, Marina Gritsenko, Gina M. Many, Karina H. Nakayama

**Affiliations:** ^1^ Department of Biomedical Engineering Oregon Health and Science University Portland Oregon USA; ^2^ Biological Sciences Division Pacific Northwest National Laboratory Richland Washington USA; ^3^ Department of Orthopaedics and Rehabilitation Oregon Health and Science University Portland Oregon USA

**Keywords:** aging, inflammation, muscle regeneration, phosphoproteomics, stress response, volumetric muscle loss

## Abstract

Severe muscle trauma disrupts endogenous repair mechanisms and produces chronic functional deficits that are poorly defined in aging. We investigated inflammatory, molecular, and physiological responses to volumetric muscle loss in young adult and aged female mice. Cytokine profiling revealed elevated baseline inflammation in aged mice and a blunted early injury response; for example, IL‐6 increased 4.4‐fold in young versus 1.8‐fold in aged mice at day 3 post‐injury. By day 28, histological analyses revealed comparable reductions in muscle size and increased fibrosis across ages. Despite similar structural pathology, age‐dependent differences emerged in functional and molecular adaptations. Both groups exhibited persistent force deficits; however, aged muscles showed significantly altered relaxation kinetics (*p* < 0.001), suggesting dysregulated excitation‐contraction coupling. Aged mice also demonstrated altered post‐injury limb loading patterns. Global proteomics identified age‐associated enrichment of complement and antigen‐processing pathways and signatures of metabolic dysfunction (*p* < 0.05). Phosphoproteomic analysis revealed reduced basal kinase activity in aged muscle but exaggerated injury‐induced phosphorylation of Mapk1‐associated sites indicating a dysregulated stress response. Together, these findings indicate that aging muscles operate within a heightened inflammatory and perturbed kinase‐signaling environment that may impair coordinated regeneration and functional recovery following traumatic injury.

## Graphical abstract

Muscle from aged mice exhibits dysregulated inflammatory and kinase‐signaling responses and altered functional dynamics following volumetric muscle loss, despite structural pathology similar to that in young mice.

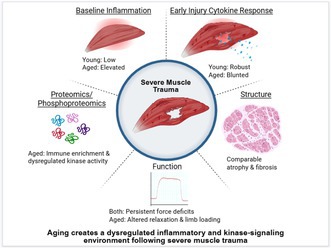

## 1 INTRODUCTION

Volumetric muscle loss (VML) is defined by the irreversible loss of skeletal muscle mass and function following traumatic injury or surgical excision of a critically sized portion of muscle (Garg et al., 2015). In contrast to minor injuries, which regenerate through the coordinated actions of endogenous immune cells, satellite cells, and extracellular matrix (ECM) remodeling, VML overwhelms these intrinsic repair programs, leading to chronic functional deficits. These functional deficits arise from two distinct but interacting components: the irreversible loss of contractile muscle tissue, which directly reduces force‐generating capacity, and maladaptive molecular and cellular responses within the remaining muscle that further impair regeneration and functional performance. VML pathology reflects a convergence of immune dysregulation, fibrotic remodeling (Hymel et al., 2023), metabolic disturbances (Dalske et al., 2021), and secondary denervation (Sorensen et al., 2021). Regenerative strategies, including stem cell transplantation, engineered biomaterials, and gene therapies, show promise for restoring muscle structure and function, yet their translation is hindered by an incomplete mechanistic understanding of VML pathophysiology.

Recent transcriptomic and proteomic studies have begun to map the molecular mechanisms which drive VML pathology. In a preclinical mouse model, longitudinal RNA sequencing and proteomics (days 3, 7, 14, and 21 post‐injury) identified multiple dysregulated programs that failed to return to pre‐injury baselines within 21 days of injury. These molecular signatures mirrored cell‐ and tissue‐level pathologies, including sustained upregulation of extracellular matrix remodeling and inflammatory signaling, coupled with persistent downregulation of mitochondrial and metabolic functions. Notably, this study proposed the transcription factor SP1, which is involved in a multitude of cellular processes, as a potential novel VML regulatory node (Jain et al., 2025). Complementary multi‐omics work in a canine VML model reinforced these findings, noting persistent dysregulation of immune‐ECM crosstalk as a central barrier to regenerative healing. Collectively, these studies establish a spatiotemporal molecular framework to further understand why VML injuries remain non‐reparative. Yet both regenerative therapy development and mechanistic pathology studies are limited by their reliance on young, highly regenerative systems, creating a critical barrier to translation. Building on this foundation, future investigations must incorporate aged models to better reflect the diverse patient populations affected by VML.

While many VML injuries occur in young military populations, high‐energy civilian trauma and surgical resections (e.g., soft tissue sarcoma excision) can affect individuals of all ages. Aging alone alters muscle function, maintenance, and regenerative capacity, with both omics‐based and tissue‐level studies identifying changes in contractile machinery, inflammatory dynamics, ECM composition, and satellite cell activity, among others (Distefano & Goodpaster, 2018; Han et al., 2021; Miljkovic et al., 2015). These age‐associated alterations are translationally relevant, as they directly influence the effectiveness of regenerative therapies. Despite the success of tissue engineered approaches in restoring muscle function in young injury models, these treatments fail to achieve comparable outcomes in aged systems (Habing et al., 2024; Kim et al., 2019). By identifying pathways that impair regeneration in aged muscle, studies such as this one may reveal targets for adjuvant therapies that could increase the efficacy of VML repair strategies. Thus, understanding how the “dual insult” of VML and aging jointly shapes molecular and physiological responses is essential for designing or adapting therapies that promote effective regeneration across the lifespan.

The present study addresses this gap by profiling both the molecular and functional consequences of a surgically induced VML injury in young adult and aged mice. Longitudinal serum cytokine profiling was paired with terminal global and phospho‐muscle proteomics to capture both the transient inflammatory dynamics and the persistent molecular programs that diverge with age. These molecular signatures were examined alongside assessments of muscle function, linking age‐dependent changes in protein expression with physiological outcomes such as contraction kinetics. This integrative approach establishes a systems‐level framework for understanding how aging modulates VML pathology and identifies potential molecular targets for candidate regenerative therapies targeting the restoration of muscle function following severe traumatic injury across the lifespan.

## 2 MATERIALS AND METHODS

### 2.1 Experimental design and injury model

**FIGURE 1:**
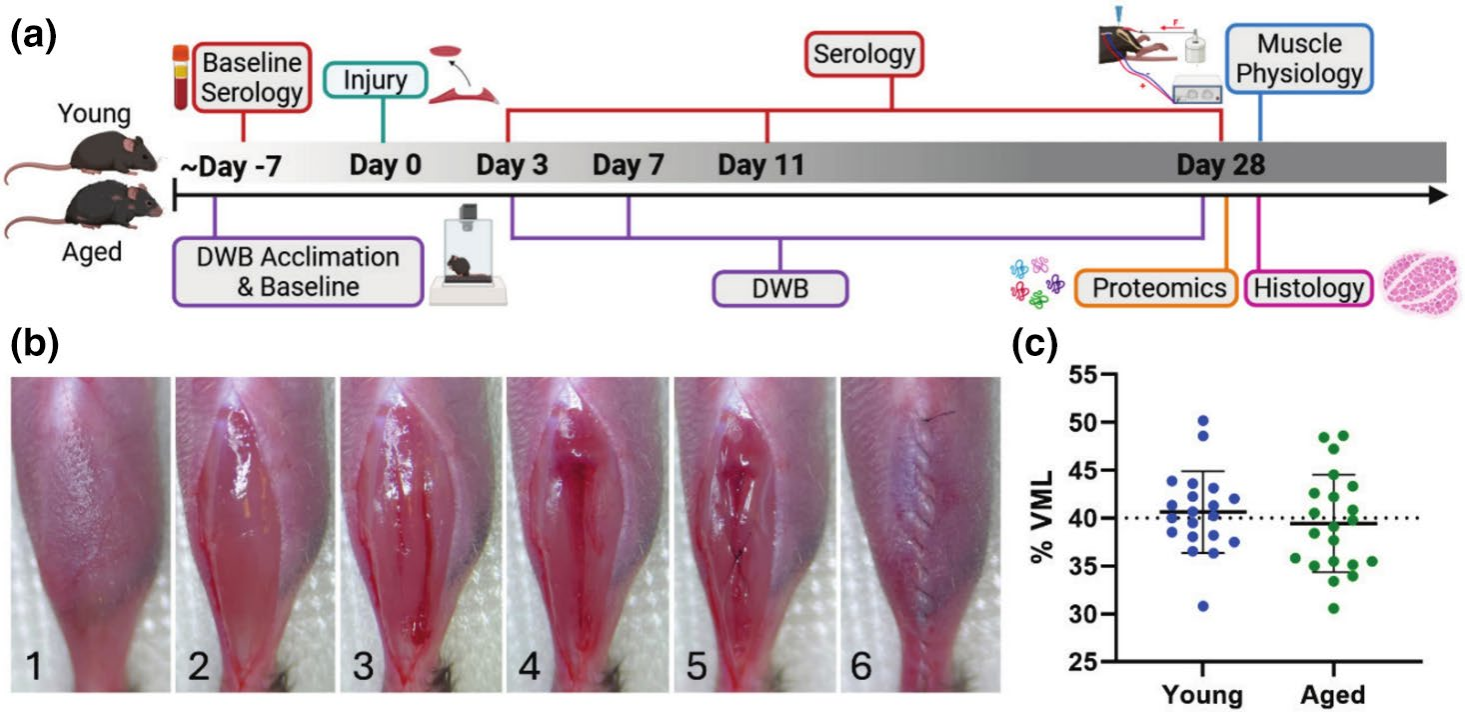
Characterization of young and aged murine volumetric muscle loss model. (a) Schematic of experimental design. Young and aged mice underwent baseline assessments prior to surgical induction of a tibialis anterior (TA) volumetric muscle loss (VML) injury. Longitudinal outcomes included serology‐based cytokine profiling (days 3, 11, 28) and dynamic weight bearing (days 3, 7, 28) to non‐invasively assess limb use and pain. Terminal outcomes at 28 days post‐injury included TMT‐labeled proteomics, histological analyses, and functional assessments using in situ muscle physiology. (b) Intraoperative images of the VML procedure showing: (1) unperturbed hindlimb, (2) skin incision exposing the TA muscle, (3, 4) 40% partial‐thickness TA muscle ablation, (5) muscle closure, and (6) skin closure. (c) Validation of relative VML defect size between young (blue) and aged (green) mice (*N* ≥ 20). Significance was determined as *p* < 0.05 using a two‐tailed, two‐sample homoscedastic *t*‐test. Shown are mean ± SD.

Age‐related physiological, molecular, and structural differences in muscle regeneration following volumetric muscle loss (VML) were characterized in young adult (8–10 weeks, *N* = 20) and aged (80–96 weeks, *N* = 21) female C57BL/6 mice (Jackson Laboratories, Figure 1a). Female mice were selected during development of a broader study that included a treadmill‐based exercise intervention, as aged females completed the exercise protocol more consistently than males. Sex was therefore held constant across all groups. The analyses reported here focus on age‐related responses to VML in non‐exercised female mice. All animal procedures were approved by the Oregon Health & Science University Institutional Animal Care and Use Committee (Protocol Number: TR01_IP00002839). Mice were group‐housed at a maximum of five per cage in standard cages (Thoren Caging Systems Inc., 7.70 × 12.17 × 5.25 inches) under a 12:12‐h light–dark cycle, with ad libitum access to food (PicoLab® Laboratory Rodent Diet, #5L0D) and water.

The VML model was adapted from previously described protocols (Alcazar et al., 2020; Habing et al., 2024; Nakayama et al., 2018; Nakayama et al., 2019; Tan et al., 2025). Mice were anesthetized under 3% isoflurane in 100% oxygen at a flow rate of 1 L/min. Hindlimb hair was removed with a depilatory cream, and the surgical field was sequentially disinfected with iodine and alcohol. Animals received subcutaneous injections of enrofloxacin (5 mg/kg, MWI, 119838), extended‐release buprenorphine (1 mg/kg, Wedgewood, BUPREN‐INJ010VC), and warmed sterile saline (0.9% sodium chloride, 1 mL).

The injury model consisted of a partial thickness defect which was surgically created by excising 30%–50% of the left tibialis anterior (TA) muscle, with the right TA serving as an uninjured contralateral control (Figure 1b,c). Injury size was estimated by calculating the ratio of excised tissue mass at the time of surgery to the post‐mortem mass of the contralateral TA. This defect size was selected to produce a sustained functional deficit and minimize spontaneous recovery, consistent with prior larger‐defect TA VML models (Quarta et al., 2017; Vega‐Soto et al., 2020). The injury margins were closed with cruciate 8–0 nylon sutures, and skin closure was performed with a continuous suture of the same material.

Regenerative outcomes were assessed longitudinally with serum cytokine profiling and dynamic weight bearing, while terminal analyses included histology, proteomics, and in situ muscle physiology. VML size, dynamic weight bearing (DWB), and cytokine measurements were collected from all mice. DWB was collected at baseline, day 3, day 7, and day 28. Based on cytokine profiling schedules, animals were subsequently allocated into separate cohorts for terminal outcome measures. A subset of mice was evaluated only at baseline and day 3 for VML size, DWB, and cytokine profiling before removal from the study. Of the remaining animals, one cohort was profiled at baseline, day 3, day 11, and day 28 and used for proteomic analyses, whereas a second cohort was assessed at baseline, day 11, and day 28 and used for muscle physiology testing and histology. Differences in serum collection scheme were not expected to influence other outcome measures. Exact sample sizes for each assay and time point are reported in the corresponding Methods sections.

### 2.2 Serum collection and cytokine profiling

Serum samples were collected longitudinally at baseline (Young = 19, Aged = 20) and at 3 (Young = 8, Aged = 8), 11 (Young = 10, Aged = 12), and 28 (Young = 10, Aged = 12) days post‐surgery. Mice were anesthetized with 3% isoflurane, and 70–80 μL of blood was obtained per session using a non‐heparinized capillary tube (Fisher Scientific) inserted into the medial canthus of alternating eyes. Samples were left to clot at room temperature for ≥30 min before centrifugation at 2500 × g for 15 min at 4°C. Serum was collected and stored at −80°C until analysis.

Cytokine levels were measured using the Milliplex MAP Mouse Cytokine/Chemokine Magnetic Bead Panel (Millipore Sigma, MCYTMAG‐70K‐PX32) on a Luminex 200. Median fluorescent intensity (MFI) values and bead counts were recorded. All samples were run at a 1:8 dilution, as determined from pilot testing. Data were analyzed in R (v 4.5.1). Cytokines with average bead counts <50 and/or average MFI <20 were excluded from further analysis. One aged mouse was excluded due to having average cytokine MFI values 3.5 standard deviations above the group mean.

MFI values were log_2_‐transformed and batch‐corrected using a design matrix that preserved treatment and timepoint effects using the limma R package. Partial least squares discriminant analysis (PLSDA) was conducted using the ROPLS package. Models were built with two latent variables (LV1 and LV2) to maximize group separation. LV score plots were used to visualize separation based on age and timepoint while LV loading plots identified cytokines most strongly contributing to each latent variable. Cytokines were *z*‐scored for heatmap visualization.

### 2.3 Dynamic weight bearing analysis

Longitudinal, non‐invasive dynamic weight bearing (DWB) measurements were obtained at baseline (Young = 20, Aged = 22) and at 3 (Young = 20, Aged = 22), 7 (Young = 10, Aged = 12), and 28 (Young = 10, Aged = 12) days post‐surgery using the BioSeb Dynamic Weight Bearing 2.0 chamber, with weight distribution recorded via DWB2 software. Acquisition settings included an immobility threshold of 700 ms, a neighboring pixels weight threshold of 0.7 g, a minimum paw size of 2 pixels, and a central pixel weight threshold of 0.9 g. To minimize behavioral artifacts, data were collected prior to other procedures and environmental stimuli were reduced. Animals were acclimated to the chamber a day prior to baseline data collection for 10 min. For subsequent sessions, mice underwent a 1‐min re‐acclimation period followed by 5 min of recording, with at least 2 min of total validated data required for analysis. Only postures classified as mobile were quantified to capture active limb use. The following parameters were calculated: time mobile, percent body weight borne on the injured leg (rear left), rear left pressure (paw weight normalized to paw area), percent time the injured leg was not weight bearing, front‐to‐rear weight ratio, and rear left‐to‐right weight ratio.

### 2.4 In situ muscle functional testing

On day 28 post‐injury, TA muscle force and contractile dynamics were assessed using in situ muscle physiology (Young = 6, Aged = 7; in each age group, at least one injured leg was non‐responsive during testing, likely due to procedural trauma during setup). Mice were anesthetized with 1%–3% isoflurane and hindlimb fur was removed. The TA muscle was exposed by removing the overlying skin and fascia, followed by isolation of the muscle through severance of the distal TA tendon and surgical separation from adjacent muscles. The distal tendon was secured to a K1000 force transducer (Harvard Apparatus) with a 6–0 silk suture. A custom nerve cuff was positioned on the deep peroneal nerve, and the leg was stabilized via a needle inserted through the knee joint. The TA was stimulated using a GRASS S48 stimulator.

During testing, animal body temperature was regulated with a heat lamp, and the TA muscle was regularly moistened with warmed saline. A passive tension of 5 cN and pulse duration of 0.5 ms were maintained throughout testing. A voltage sweep was performed at a frequency of 140 Hz, to determine the optimal stimulation voltage for maximal isometric contraction, followed by a frequency sweep from 60 to 140 Hz at this optimal voltage. One‐minute rest intervals were provided between contractions. Contractions were recorded as output voltages using LabChart (v8.1.19).

Force traces were analyzed with a custom MATLAB (2023b) script. The script detected contraction peaks automatically, segmented force responses, converted voltage output to force (Equation 1), applied baseline correction, and calculated physiological metrics including peak force, rate of force development, and contraction duration. Force signals were smoothed with a moving‐average filter before peak detection and derivative calculations. Maximum contraction and relaxation rates were defined as the extrema of the first derivative of force normalized to the maximum force of the respective contraction. Peak force was taken from the highest recorded contraction regardless of stimulation frequency, while contraction dynamics were compared at 100 Hz due to their strong frequency dependence (Figure S1A–C). Contraction duration was measured at 10% of peak force to minimize baseline noise effects.

(1) $$\begin{array}{c} \text{Force}=\left(\text{Maximum Output}\;\left(V\right)-\text{Baseline Output}\;\left(V\right)\right)\\ \;*50\;\frac{\text{gram force}}{\text{volt}}*0.0098\frac{\text{Newton}}{\text{gram force}} \end{array}$$

### 2.5 Histological analysis of muscle morphometrics

Following completion of muscle physiology testing, mice were euthanized by 1% carbon dioxide exposure followed by cervical dislocation. The left (injured) and right (contralateral control) TA muscles were excised, weighed, and fixed overnight at 4°C in 0.2% paraformaldehyde under agitation. Fixed muscles were equilibrated in 20% sucrose and embedded in optimal cutting temperature compound prior to snap freezing. Transverse cross sections (10 μm thick) from three mice per age group were stained with either standard hematoxylin & eosin (H&E) or Masson's Goldner Trichome protocols. For the latter, an additional overnight fixation in Bouin's solution was included to enhance stain intensity. Brightfield images were acquired at 4× magnification using a Keyence BZ‐X1000 microscope, with image stitching performed as needed in ImageJ (v1.54i) using the Pairwise Stitching plugin. Collagen deposition was quantified as percent area using a custom MATLAB (2023b) script for background subtraction in combination with ImageJ's Color Deconvolution tool to threshold collagen‐positive regions relative to total muscle area.

### 2.6 Proteomics data collection, processing, and analysis

On day 28 post‐injury and following euthanasia via 1% carbon dioxide overdose and subsequent cervical dislocation, the left (injured) and right (contralateral control) TA muscles were excised, weighed, and briefly rinsed in HALT protease solution (ThermoFisher) prior to flash freezing in liquid nitrogen (Young = 4, Aged = 5, both TA muscles). Tissues were stored at −80°C prior to analysis via a standardized protocol described below (Sanford et al., 2022).

#### 2.6.1 Tissue lysis and protein digestion

Muscle samples were homogenized in freshly prepared, ice‐cold lysis buffer 8 M urea, 50 mM Tris pH 8.0, 75 mM sodium chloride, 1 mM EDTA, 2 μg/mL aprotinin (Sigma‐Aldrich), 10 μg/mL leupeptin (Roche), 1 mM PMSF in EtOH, 10 mM sodium fluoride, 1% phosphatase inhibitor cocktails 2 and 3 (Sigma‐Aldrich), 10 mM Sodium Butyrate, 2 μM SAHA, and 10 mM nicotinamide using a tissue tearer. Once homogenized, samples were incubated on a thermomixer (15 min, 4°C, 800 rpm), vortexed for 10 s, incubated again with the same settings, and centrifuged for 10 min at 4°C and 18,000 × g to remove debris. Protein concentrations from the resulting supernatants were determined by bicinchoninic acid (BCA) assay, and lysis buffer was used to adjust all samples to a final protein concentration of 8 μg/μL.

Samples were reduced with 5 mM dithiothreitol (DTT, Sigma‐Aldrich) for 1 h in a thermomixer set to 37°C and 800 rpm, and reduced cysteine residues were alkylated with 10 mM iodoacetamide (IAA, Sigma‐Aldrich) for 45 min in a thermomixer set to 25°C and 800 rpm. Samples were diluted 3‐fold with 50 mM Tris–HCl, pH 8.0, and initially digested with Lys‐C (Wako) at a 1:50 enzyme: substrate ratio for 2 h in a thermomixer set to 25°C and 800 rpm. Following the initial digest, trypsin (Promega) was added at a 1:50 enzyme: substrate ratio, followed by a 14 h incubation at 25°C and 800 rpm. Digestions were quenched by acidifying the solution to 1% formic acid, after which samples were centrifuged for 15 min at 1500 × g. The resulting peptides were desalted using C18 solid phase extraction (SPE) cartridges (Waters Sep‐Pak).

#### 2.6.2 Tandem mass tag (TMT) labeling and HPLC fractionation

Peptide concentrations from the cleaned samples were determined by BCA assay, and 250 μg of peptide from each sample was aliquoted into fresh tubes and dried completely in a vacuum centrifuge. Sample were resuspended in 250 μL of 250 mM HEPES buffer (final peptide concentration of 5 μg/μL). Tandem Mass Tag (TMT) 18‐plex isobaric label reagents (ThermoFisher) were resuspended in anhydrous acetonitrile at a concentration of 40 μg/μL, and 500 μg of a unique TMT label was added to each of the 18 samples (2:1 TMT label: peptide ratio). Labeling was allowed to proceed for 1 h in a thermo‐mixed at 25°C and 600 rpm. Following a QC check of TMT labeling efficiencies, reactions were quenched with 5% hydroxylamine, and all samples were combined and desalted using C18 SPE columns (Waters Sep‐Pak).

The combined TMT multiplex sample was then fractionated into 96 fractions by high‐pH reversed phase chromatography using a 4.6 mm ID × 250 mm length Zorbax 300 Extend‐C18 column (Agilent). Samples were loaded on the column in buffer A (4.5 mM ammonium formate in 2% acetonitrile) and eluted off the column using a gradient of 0%–60% buffer B (4.5 mM ammonium formate in 90% acetonitrile) at a flow rate of 1 mL/min for 96 min. The 96 fractions were then concatenated into 24 fractions using a scheme of fraction 1 + 25 + 49 + 73; fraction 2 + 26 + 50 + 74; fraction 3 + 27 + 51 + 75; etc. (Wang et al., 2011). 5% of each fraction was removed for global proteomics analysis, and the remaining 95% was further concatenated to 12 fractions for phosphopeptide enrichment. Prior to LC–MS/MS analysis, the global fractions were dried in a vacuum centrifuge and resuspended in 3% acetonitrile + 0.1% FA at a concentration of 0.1 μg/μL.

#### 2.6.3 Phosphopeptide enrichment

Phosphopeptide enrichment was performed using the Agilent AssayMap Phosphopeptide enrichment platform with Fe(III)‐NTA cartridges. First, peptide fractions were resuspended in 200 μL of 80% acetonitrile (ACN)/0.1% TFA and then centrifuged at 6000 × g for 5 min to remove insoluble material. Samples were transferred to polypropylene 96‐well plates. Priming buffer for the cartridges consisted of 50% ACN/0.1% TFA, 80% ACN/0.1% TFA was used as the equilibration buffer and internal cartridge wash buffer, and the elution buffer was 1% aqueous ammonia. Default protocol values were used for phosphopeptide enrichment. Enriched samples were directly eluted from AssayMAP Fe(III)‐NTA cartridges into a PCR plate containing 20 μL of 10% formic acid and 6 μL of 0.02% n‐dodecyl β‐D‐maltoside. Samples were then dried in a vacuum centrifuge and reconstituted in 12 μL of 3% ACN/0.1% FA prior to LC–MS/MS analysis.

#### 2.6.4 LC–MS/MS analysis

For mass spectrometry analysis of both global and phosphoproteomic samples, online separation was performed using a Vanquish Neo UHPLC system (ThermoFisher) and a 30 cm (length) × 75 μm (inner diameter) picofrit column packed in‐house with 1.7 μm Waters Acquity BEH particles. Samples were analyzed on an Exploris 240 mass spectrometer (ThermoFisher) with MS1 scans across the 300–1800 m/z range at 60,000 resolution, a normalized AGC target of 300%, and maximum injection time set to auto. MS2 scans of the 20 most abundant ions were performed at 45,000 resolution with a 0.7 m/z isolation window, 30% normalized collision energy, and AGC target and maximum injection time set to auto. Dynamic exclusion window was set to 30 s.

#### 2.6.5 Proteomics data processing

The obtained MS/MS spectra were searched using the MS‐GF+ tool against a mouse reference proteome downloaded from UniProt in March 2023 for peptide sequence identification (Kim & Pevzner, 2014). Carbamidomethylation on cysteine residues and TMT‐11 modifications on lysine residues and the N termini were set as fixed modifications, with oxidation on methionine residues as a dynamic modification. For phosphoproteomics, phosphorylation on serine, threonine, and tyrosine residues was set as dynamic modifications. Localization of phosphorylation modifications was performed using the Ascore algorithm (Beausoleil et al., 2006). For identified peptides, the TMT reporter ion intensities (RII) were extracted using MASIC (Monroe et al., 2008). Contaminant proteins were removed, and data from all fractions (24 fractions for global proteomics, 12 fractions for phosphoproteomics) were aggregated to the unique protein (global proteomics) or phosphosite (phosphoproteomics) level using the PlexedPiper package in RStudio (Petyuk, 2025). The mean RII value was calculated for each feature (unique protein or phosphosite), and the RII values for each experimental sample were then divided by this value and log_2_‐transformed to generate log_2_(RII/mean) values for each feature in each sample. Within each sample, data were normalized by median‐centering according to the central tendency method.

#### 2.6.6 Statistical analysis of proteomics data

Differential analysis of global proteomics and phosphoproteomics data were carried out with the limma R/Bioconductor package (Ritchie et al., 2015). A no‐intercept model was fit where the sole predictor was a factor with levels for each combination of age (Y: Young; A: Aged) and hindlimb (R: right, uninjured; L: left, injured). Contrasts were specified to identify differences due to age separated by the presence of muscle injury (A.R–Y.R, A.L–Y.L) and to identify age‐specific differences that were due to muscle injury (Y.L–Y.R, A.L–A.R). We first examined multi‐dimensional scaling (MDS) plots–distances between samples approximate their average log_2_(fold‐change)–and observed one or more samples from the experimental groups that separated from the rest. To address this separation, subject‐level quality weights–calculated with the arrayWeights function–were incorporated into the linear model to down‐weight those pairs of samples with high residual variance (Ritchie et al., 2006). Additionally, due to the repeated measures design where each mouse has a sample taken from both left and right hindlimbs, the average within‐mouse sample correlation was estimated with the duplicateCorrelation function and included in the model fit, which was constructed with the lmFit function (Smyth et al., 2005). Lastly, an Empirical Bayes approach was used to squeeze the residual variances of the proteins or phosphorylation sites toward a global mean–variance trend with the Bayes function; this trend was made robust to the presence of hypo‐ or hyper‐variable features (i.e., the function arguments trend and robust were set to TRUE). This approach has the effect of increasing the residual degrees of freedom and, therefore, power to detect differences. *p*‐values were adjusted separately by contrast with the method of Benjamini and Hochberg to control the false discovery rate (FDR), and proteins or phosphorylation sites with adjusted *p*‐values <0.1 were considered statistically significant.

#### 2.6.7 Analysis of molecular signatures in proteomics data

Gene sets from the Reactome and Gene Ontology subcollections of the mouse Molecular Signatures Database (MSigDB) (Castanza et al., 2023; Liberzon et al., 2011; Liberzon et al., 2015), gene sets from the Pathway Interaction Database (PID) subcollection of the human MSigDB, and human gene sets from the CellMarker 2.0 database were selected for testing (Hu et al., 2023; Schaefer et al., 2009). The human gene sets were converted to mouse equivalents with the biomaRt R/Bioconductor package, and gene sets with at least 10 genes that overlapped with the proteomics data were retained.

For phosphoproteomics, kinase sets were formed by grouping phosphosites by their known kinases according to information in the “Kinase_Substrate_Dataset” file from PhosphoSitePlus (v6.7.1.1) (Hornbeck et al., 2015). In addition, we tested sets of phosphorylation sites from the Post‐Translational Modification Signatures Database (PTMsigDB) (Krug et al., 2019). Only those kinase sets or PTMsigDB signatures with at least 3 phosphosites that overlapped with the phosphoproteomics data were retained.

The analysis of gene and kinase sets was carried out with the pre‐ranked Correlation Adjusted MEan RAnk gene set test (CAMERA‐PR)—a modified two‐sample *t*‐test that accounts for correlation within sets to better control the type I error rate—using the cameraPR function from limma (Wu & Smyth, 2012). The feature‐level moderated *t*‐statistics from the limma differential analysis results were converted to *z*‐scores and used as input for CAMERA‐PR. The analysis of signatures from PTMsigDB was carried out with the fast variant of Single‐Sample Gene Set Enrichment Analysis (ssGSEA) from the fast.ssgsea R package (https://github.com/pnnl/fast.ssgsea), where the specific combination of ssGSEA and PTMsigDB is known as Post‐Translational Modification Signature Enrichment Analysis (PTM‐SEA). A total of 10,000 permutations were used to estimate normalized enrichment scores (NES) and calculate exact permutation *p*‐values.

### 2.7 Statistical analysis

Proteomic analyses are described separately. Cytokine profiling data were statistically analyzed in R (v4.5.1) and while all other analyses were performed in GraphPad Prism (v10.6.1). Two‐way ANOVA with Sidak (cytokine, DWB) or Fisher's LSD (histology, physiology) post hoc tests were used to assess the effects of age and timepoint, or age and injury status. A summary of all two‐way ANOVA outcomes, including main and interaction effects, is provided in Table S1. For comparison of VML size, a two‐tailed, two‐sample homoscedastic *t*‐test was applied. Statistical significance was defined as *p* < 0.05 (*), *p* < 0.01 (**), *p* < 0.001 (***), and *p* < 0.0001 (****). Data are presented as mean ± standard deviation.

## 3 RESULTS

### 3.1 Volumetric muscle loss model characterization

To evaluate how age‐associated molecular and physiological differences influence skeletal muscle regeneration after severe injury, a unilateral partial thickness defect was created in the TA muscle of young and aged mice (Figure 1a,b). A defect exceeding 20% of a muscle's mass is considered representative of volumetric muscle loss (Grogan & Hsu, 2011). For this study, a target of 40% loss was selected to produce a reliably non‐regenerative defect and to increase sensitivity for detecting age effects across molecular and functional endpoints. Injury size was validated by comparing the mass of the excised TA muscle at surgery with the mass of the contralateral uninjured muscle harvested on day 28 post‐surgery. Defect size ranged from 30.6% to 50.2%. Mean loss approximated the 40% target, at 40.6% ± 4.3% in young mice and 39.4% ± 5.1% in aged mice, with no statistical difference between age groups (*p* = 0.42, Figure 1c).

### 3.2 Systemic cytokine and chemokine dynamics

**FIGURE 2:**
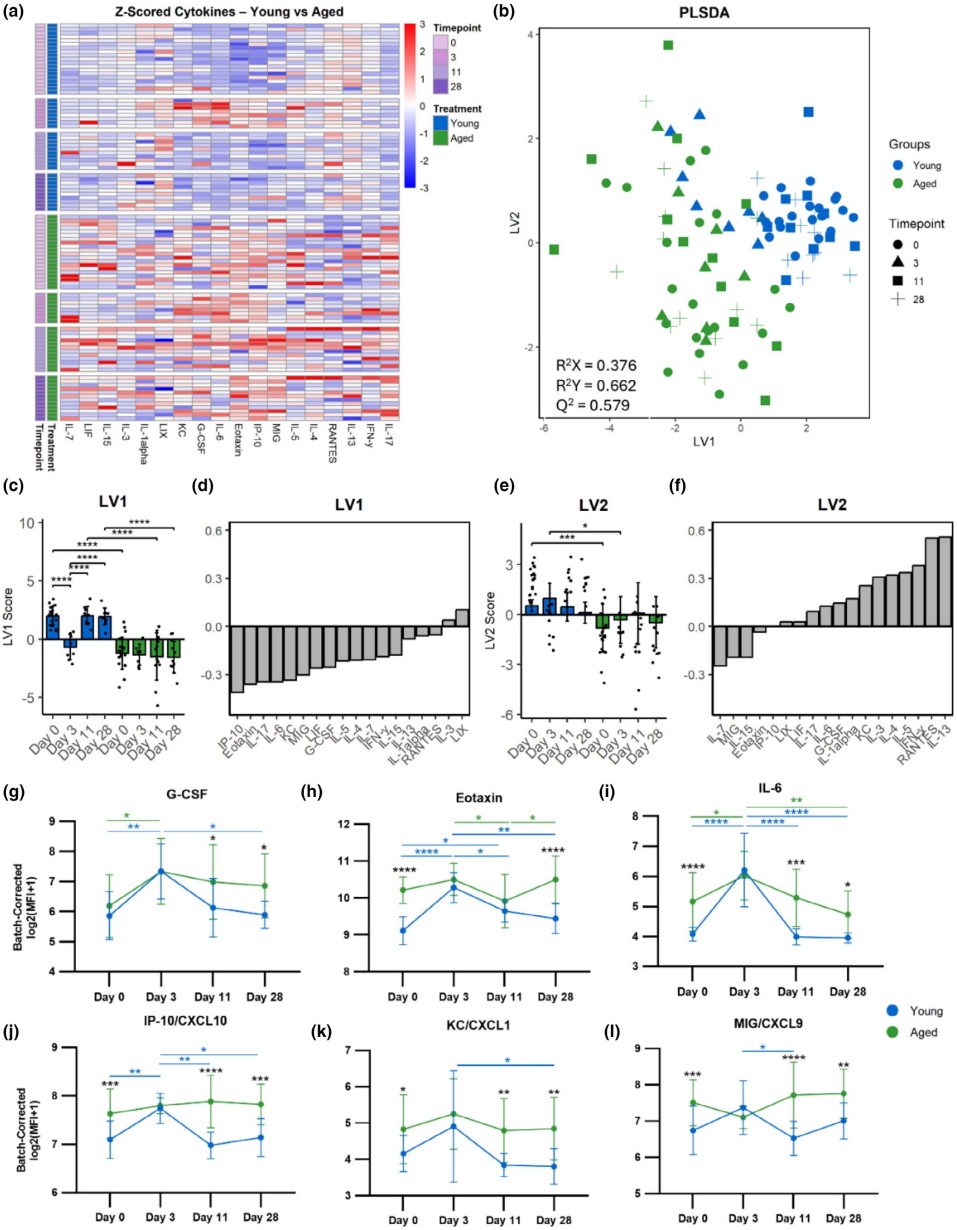
Age‐dependent cytokine profiles following VML injury. (a) Heatmap of cytokine expression (columns) reported as *z*‐scored median fluorescent intensity (MFI) values at baseline (Day 0, *N* ≥ 19) and post‐injury (Days 3, *N* = 8; Day 11, *N* ≥ 10; Day 28, *N* ≥ 10). Red indicates higher expression and blue indicates lower expression. (b) Partial least squares discriminant analysis (PLSDA) of cytokine profiles, with each point representing a sample plotted by latent variable (LV) scores. Samples are colored by age (Young = blue; Aged = green) and shaped by timepoint. (c, e) LV1 and LV2 score distributions by age and timepoint. (d, f) LV loading plots showing cytokines with the strongest contributions to LV1 (d) and LV2 (f). (g–l) Representative cytokine time courses in young (blue) and aged (green) mice, shown as log_2_‐transformed MFI values. Significance was determined by two‐way ANOVA with Sidak post hoc test with *p* < 0.05 (*), *p* < 0.01 (**), *p* < 0.001 (***), and *p* < 0.0001 (****). Black asterisks represent significance between age groups at a single timepoint. Colored asterisks represent significance between timepoints within an age group. Shown are mean ± SD.

To determine how age altered the post‐injury systemic inflammatory response, serum cytokines were measured using a Luminex multiplex immunoassay in young and aged mice at baseline and at days 3, 11, and 28 post‐injury. Eighteen analytes passed quality control (Bead Count >50 and MFI >20). Analysis of log_2_ transformed *z*‐scored cytokine profiles showed higher baseline levels of inflammatory cytokines in aged mice. Young mice exhibited a transient spike in cytokine levels on day 3 that was less pronounced in aged animals. Prominent elevated day 3 analytes in young mice included IL‐6, G‐CSF, Eotaxin, and IL‐5 (Figure 2a).

Partial least squares discriminant analysis (PLSDA) projected cytokines into latent variables (LVs) that maximized separation by age group, with LV1 capturing the main age effect. The PLSDA model achieved R^2^X = 0.376, R^2^Y = 0.662, and Q^2^ = 0.579, indicating that the LVs captured 37.6% of predictor variance and 66.2% of response variance with decent predicative ability (Figure 2b). Separation on LV1 was significant between age groups at all time points except day 3. Within aged mice, LV1 scores did not differ across time, while for young mice, day 3 differed from all other times (*p* < 0.0001, Figure 2c). LV1 loadings indicated that cytokines strongly associated with the separation of the aged samples (negative LV1) included IP‐10/CXCL10, Eotaxin, IL‐17, IL‐6, and KC/CXCL1, whereas IL‐3 and LIX showed modest positive loadings toward young mice (Figure 1d). LV2 accounted for residual variation with limited discriminatory value. Modest group differences were observed at day 0 (*p* < 0.001) and day 3 (*p* < 0.05), and LV2 loadings are reported for completeness (Figure 2e,f).

To examine age‐dependent cytokine dynamics in greater detail, representative analytes identified by biological relevance and LV1 loadings were evaluated individually over time. G‐CSF had similar baselines across ages and increased by roughly 2.5‐fold at day 3 in both groups. In aged mice, G‐CSF remained elevated compared to young mice through day 28 (*p* < 0.05). IL‐6 was 2.1‐fold higher at baseline in aged mice. Young mice showed a sharp peak on day 3 (4.37‐fold above baseline, *p* < 0.0001), whereas aged mice showed a smaller, broader increase (1.82‐fold, *p* < 0.05), which did not decline significantly from day 3 until day 28 (*p* < 0.01). IP‐10/CXCL10, Eotaxin, KC/CXCL1, and MIG/CXCL9 followed similar age‐dependent patterns, with aged mice maintaining chronically elevated cytokine levels and young mice exhibiting a transient day‐3 spike that rapidly returned toward baseline (Figure 2g–l), consistent with significant age × timepoint interactions (Table S1).

Together, these findings suggest that chronic inflammation in aged mice attenuates acute cytokine responses and disrupts their normal resolution after injury. These dynamics are critical determinants of the regenerative cascade, influencing immune activation, satellite cell recruitment, and subsequent tissue repair. Translationally, these results highlight the inflammatory milieu as a potential therapeutic target that could be modulated in parallel with myogenic interventions to improve regeneration in aged populations.

### 3.3 Histological assessment of muscle area and fibrosis

**FIGURE 3:**
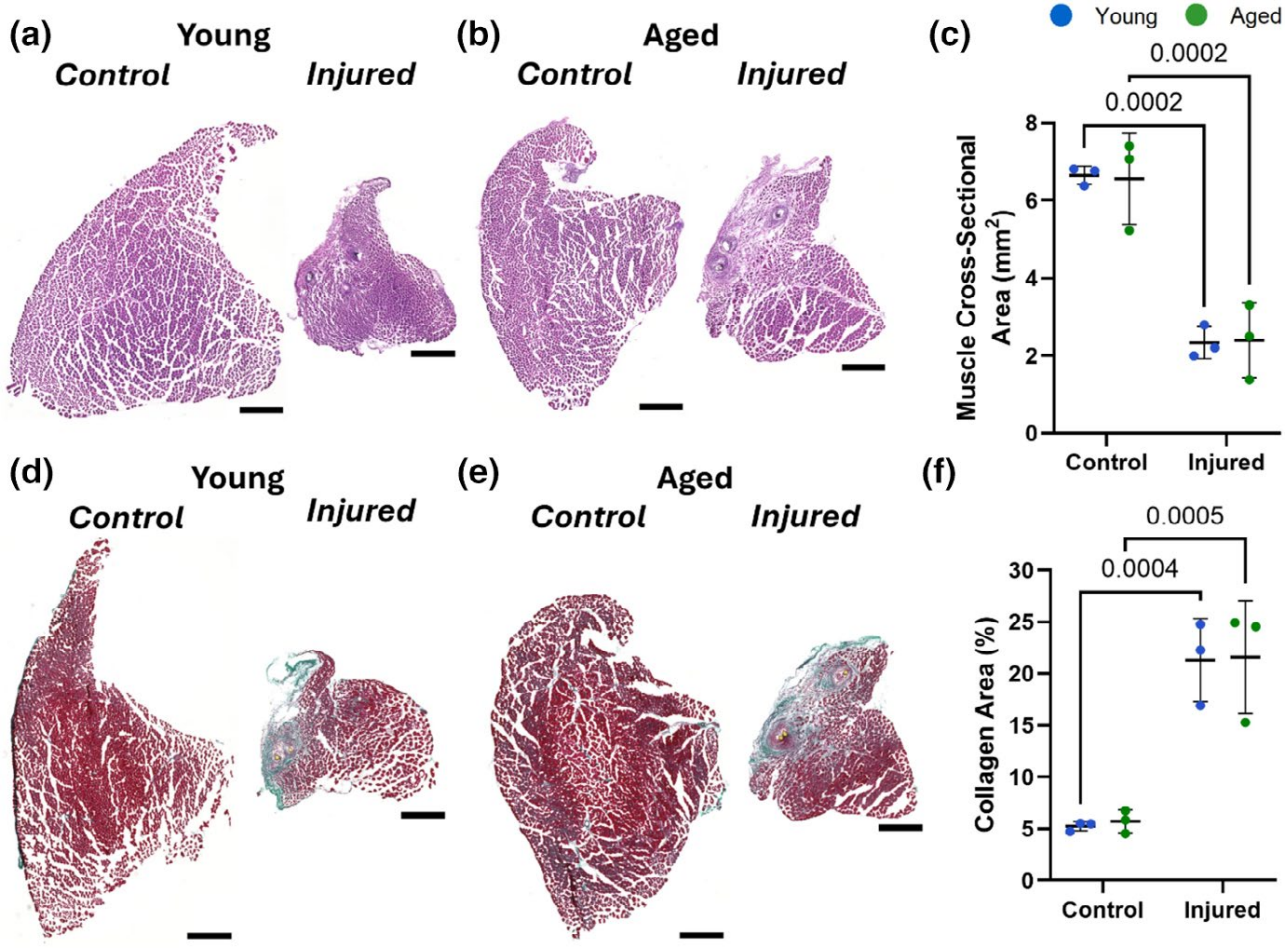
Age‐independent TA muscle morphometrics and fibrosis following VML injury. (a, b) Representative Hematoxylin & Eosin‐stained transverse sections of contralateral control (left) and injured (right) TA muscles from young and aged mice. (c) Cross‐sectional areas of control and injured TA muscles from young (blue) and aged (green) mice (*N* = 3). (d, e) Representative Masson's Goldner‐stained transverse sections of contralateral control (left) and injured (right) TA muscles from both age groups. (f) Percent collagen‐positive area in control and injured TA muscles relative to total muscle size in young (blue) and aged (green) mice (*N* = 3). Scale bars: (a, b, d, e) 500 μm. Significance was determined by two‐way ANOVA with Fisher's LSD post hoc test. Shown are mean ± SD.

On day 28 after injury, uninjured contralateral controls and injured TA muscles were excised and evaluated for muscle size based on cross‐sectional area and for collagen deposition. Hematoxylin & Eosin staining showed no age‐related difference in control muscle size (Figure 3a–c). The mean cross‐sectional area of the uninjured controls was 6.12 ± 0.28 mm^2^ in young mice and 6.16 ± 1.12 mm^2^ in aged mice. Despite 28 days of recovery, the 40% VML defect produced a 2.75‐ to 3‐fold reduction in muscle area relative to the contralateral controls (*p* < 0.001). Injured muscles averaged 2.05 ± 0.49 mm^2^ in young mice and 2.23 ± 0.92 mm^2^ in aged mice (*p* = 0.92, Figure 3c). Collagen deposition quantified by Masson‐Golder Trichrome staining on day 28 also did not differ between age groups (Figure 3d,e). In uninjured controls, collagen occupied 4.5%–6.7% of the muscle cross‐sectional area. Following injury, collagen content increased by approximately 4‐fold (*p* < 0.001), reaching up to 25% of muscle area (Figure 3f). Collectively, reductions in muscle size and increases in collagen deposition were age‐independent, indicating no differences in gross tissue loss or fibrosis following VML, with no significant age × injury interaction effects detected (Table S1).

### 3.4 Temporal assessment of weight bearing during ambulation

**FIGURE 4:**
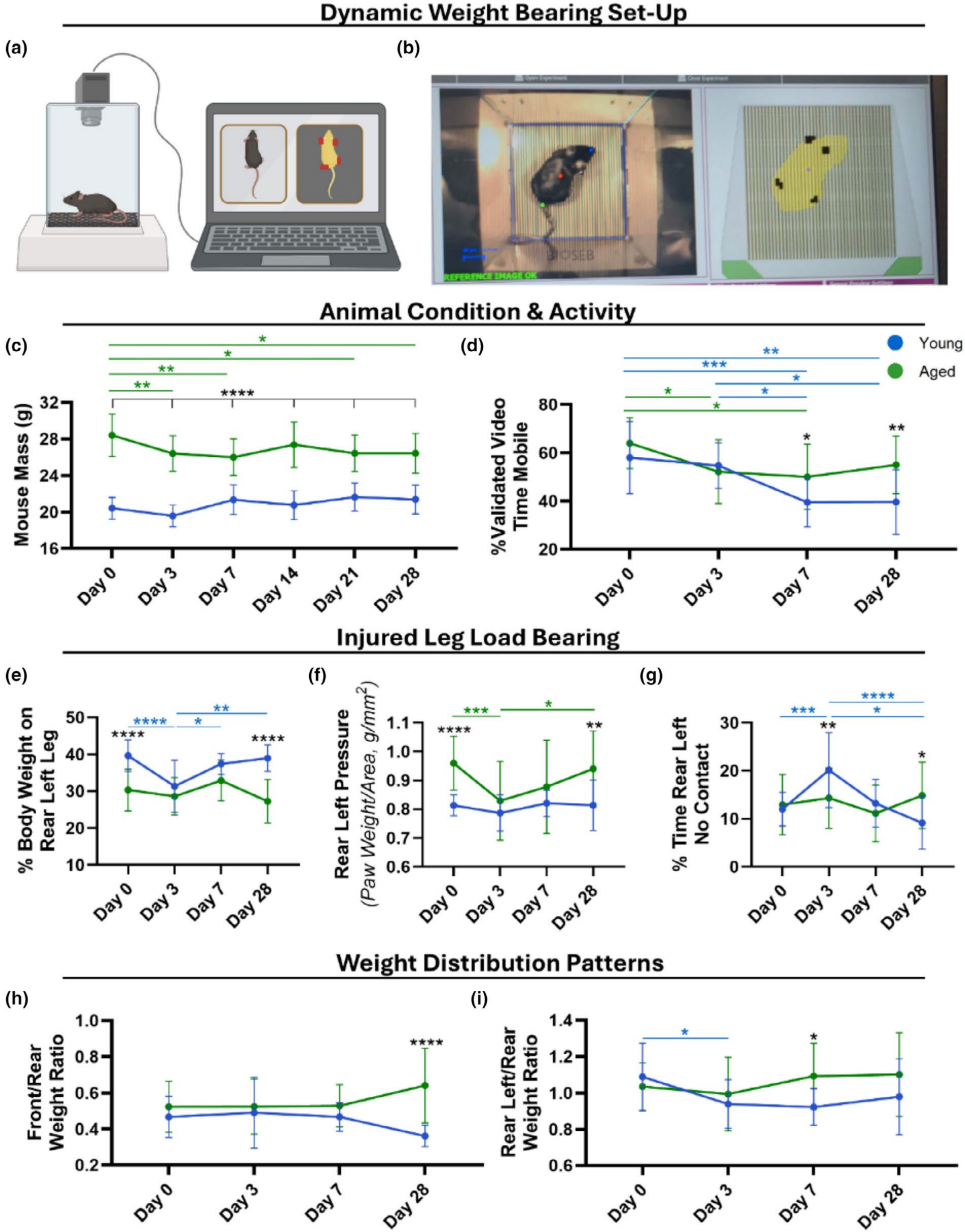
Temporal effects of VML injury on ambulatory load bearing. (a, b) Schematic (a) and photographs (b) of dynamic weight bearing system. (c) Mouse body mass at baseline (Day 0, *N* ≥20) and post‐injury (Day 3, *N* ≥20; Days 7, 14, 21, 28, *N* ≥10) in young (blue) and aged (green) mice. (d) Percent of validated recording time spent in motion over the recovery period (Days 0, 3, 7, 28) in young (blue) and aged (green) mice. (e–g) Longitudinal injured limb load‐bearing metrics, including percent body weight borne on the injured limb (e), pressure exerted on the injured limb (f), and percent validated recording time with the injured limb not in contact with the sensor (g) in young (blue) and aged (green) mice. (h, i) Whole‐body weight distribution patterns measured by front‐to‐rear limb weight ratio (h) and injured‐to‐uninjured rear limb weight ratio (i) in young (blue) and aged (green) mice. Significance was determined by two‐way ANOVA with Sidak post hoc test with *p* < 0.05 (*), *p* < 0.01 (**), *p* < 0.001 (***), and *p* < 0.0001 (****). Shown are mean ± SD.

Dynamic weight bearing was used to quantify paw‐specific load distributions over time, providing a preclinical analogue of clinical assessments of limb function and asymmetric use. When a limb is injured, quadrupedal animals such as mice typically offload weight onto their other three limbs. Thus, changes in weight distribution, contact pressure, and guarding behavior provide measurements of limb sensitivity, compensatory limb use, and functional impairments. Weight distribution across the four paws was recorded with the BioSeb Dynamic Weight Bearing 2.0 chamber, which integrates an overhead camera with a pressure sensor grid (Figure 4a,b). Body mass was recorded regularly and used to normalize weight‐bearing metrics. Aged mice were 5–8 g heavier than young mice across all timepoints. After injury, aged mice exhibited an approximately 2 g reduction in mass that was significant relative to baseline (*p* < 0.01–0.05) and remained reduced through day 28 (Figure 4c). Activity levels (% time mobile) were similar between age groups at baseline (day 0) and day 3. Activity declined over subsequent sessions, particularly in young mice, consistent with increasing habituation to the testing environment (Figure 4d).

At baseline, young mice bore 9.3% more of their body weight on their injured hindlimb than aged mice. Across the study timeline, aged mice maintained 27%–32% of their body weight on the injured limb whereas young mice showed an 8.3% decrease on day 3 with a return to baseline by day 7 (Figure 4e). Paw pressure (g/mm^2^), which incorporates both load and contact area, showed the opposite pattern. Young mice remained near 0.8 g/mm^2^ across time points while aged mice had a higher baseline (0.96 ± 0.093 g/mm^2^) and exhibited a significant reduction at day 3 (0.83 ± 0.14 g/mm^2^, *p* < 0.001) which returned to baseline by day 28 (Figure 4f). Non‐contact time of the injured limb (guarding) was about 13% of validated recording time in aged mice for all sessions. Young mice were similar at baseline, but increased the time spent guarding their injured limb to 20.12% ± 7.81% at day 3 and returned to baseline by day 7 (Figure 4g).

Changes in load distribution ratios were modest. Front‐to‐rear load ratios were comparable across time points except at day 28, when the age groups diverged (*p* < 0.0001), potentially reflecting the emergence of a longer‐term compensatory loading strategy. Rear left‐to‐right ratios were stable in aged mice while young mice showed a shift toward the contralateral hindlimb at day 3 post‐injury (0.94 ± 0.13) which was maintained for the remainder of the study (*p* < 0.05, Figure 4g,h). Overall, dynamic weight bearing analyses revealed age‐dependent differences in compensatory limb use following VML, with variations in loading that could influence subsequent muscle recovery. These differences were reflected in significant age × timepoint interactions in select load‐bearing metrics (Table S1).

### 3.5 Age and injury differentially affect muscle force and contraction kinetics

**FIGURE 5:**
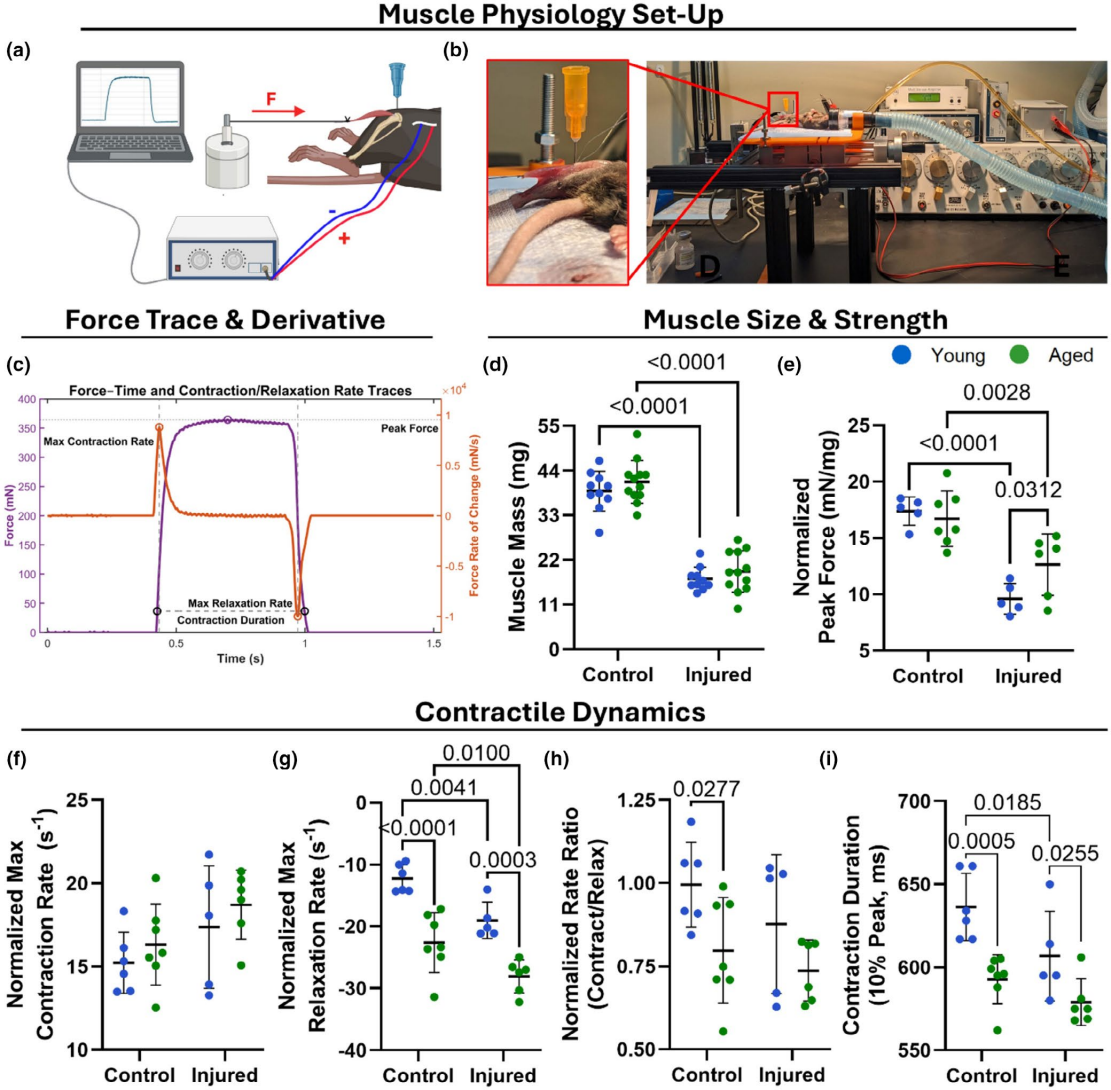
Age‐ and injury‐induced TA muscle contractile dynamics. (a, b) Schematic (a) and photographic (b) overview of in situ muscle physiology testing via peroneal nerve stimulation. (c) Representative traces of muscle contractile force (purple) and the first derivative of the force curve (orange, indicating rate of change), with annotated metrics including peak force, maximum contraction and relaxation rates, and contraction duration. (d) Mass of the contralateral control and injured TA muscles following 28 days of recovery in young (blue) and aged (green) mice (*N* ≥10). (e) Peak force of the control and injured TA muscles in young (blue) and aged (green) mice normalized to muscle mass (*N* ≥5). (f, g) Maximum rates of contraction (f) and relaxation (g) in contralateral control and injured TA muscles in young (blue) and aged (green) mice normalized to peak muscle force (*N* ≥5). (h) Contraction symmetry represented as a ratio of the normalized maximum contraction rate to relaxation rate in contralateral control and injured TA muscles in young (blue) and aged (green) mice (*N* ≥5). (i) Contraction duration measured at 10% of peak force to avoid baseline noise in contralateral control and injured TA muscles in young (blue) and aged (green) (*N* ≥5). Significance was determined by two‐way ANOVA with Fisher's LSD post hoc test. Shown are mean ± SD.

In situ muscle physiology was used to measure peak isometric force and contraction kinetics in injured and contralateral TA muscles on day 28 post‐injury (Figure 5a–c). TA wet mass was also quantified on day 28 to normalize force values and validate histological size estimates. Consistent with H&E cross‐sectional area outcomes, muscle mass was dependent on injury status, but not age. Contralateral masses averaged 38.91 ± 4.65 mg and 41.25 ± 5.26 mg in the young and aged groups, respectively (*p* = 0.26). A 40% VML injury resulted in a continued 2.2‐fold decrease in muscle mass compared to controls (*p* < 0.0001) with injured muscle from young mice averaging 17.33 ± 2.89 mg and aged averaging 19.17 ± 5.10 mg despite 28 days of recovery (*p* = 0.37, Figure 5d).

By day 28, injured TA muscles in both age groups still demonstrated less force than uninjured contralateral controls, indicating that the injury size exceeded the muscle's inherent capacity for regeneration. Peak force, normalized to muscle mass, was similar across ages in controls (Young 17.39 ± 1.26 mN/mg, Aged: 16.71 ± 2.46 mN/mg, *p* = 0.59, Video S1). Injury reduced normalized peak force by 1.81‐fold in young mice (*p* < 0.0001) and 1.32‐fold in aged mice (*p* < 0.01). Injured muscles from aged mice trended toward higher normalized peak force than young mice (Young: 9.62 ± 1.36 mN/mg, Aged: 12.63 ± 2.73 mN/mg, *p* < 0.05, Figure 5e and Video S2).

To complement peak force, tetanic contraction kinetics were assessed at 100 Hz and reported as max‐normalized rates (s^−1^), which reflect the velocity of force rise and decay independent of peak amplitude. During the contraction phase, all muscles contracted at similar rates regardless of age or injury status. However, there was a slight trend toward faster contraction in injured muscles compared to uninjured (1.15‐fold) and in aged versus young mice (1.1‐fold, Figure 5f). Unlike with the rate of contraction, the rate of relaxation is significantly impacted by both age and injury. In young mice, relaxation was 1.6‐fold faster in injured (19.01 ± 2.96 s^−1^) compared to uninjured (12.24 ± 2.25 s^−1^, *p* < 0.01) muscles. Then in aged mice, relaxation rates were 1.2‐fold faster in injured (28.06 ± 2.67 s^−1^) compared to uninjured (22.60 ± 4.85 s^−1^, *p* < 0.01) muscles. Across ages, relaxation rates were higher in aged mice regardless of injury. After injury, aged mouse muscles exceeded young by 1.5‐fold (*p* < 0.001), and uninjured muscles by 1.8‐fold (*p* < 0.0001, Figure 5g).

To further evaluate how kinetic mechanisms change with injury and age, contraction symmetry was quantified as the normalized maximum contraction‐relaxation rate ratio. Contraction symmetry was largely independent of injury status but varied with age. Muscles from young mice were approximately symmetric, with ratios within one standard deviation of 1 regardless of injury status. Aged mice showed lower ratios, averaging 0.77, consistent with relatively faster muscle relaxation compared to contraction. In contralateral controls, the symmetry ratio differed significantly between ages (*p* < 0.05, Figure 5h). Finally, contraction duration was quantified at 10% of peak force to assess whether overall contraction time differed by age or injury. Durations were tightly clustered, varying at most by ~100 ms across groups. Age had the dominant effect, with longer durations in young mice for both injured and uninjured muscles (injured: *p* < 0.05, uninjured: *p* < 0.001). Injury effects were modest, with durations being slightly longer in contralateral controls, reaching significance among young mice (*p* < 0.05, Figure 5i).

Overall, in situ muscle physiology highlighted persistent force deficits in the TA muscle after VML injury across ages, while contractile kinetics showed both age‐ and injury‐dependent effects. However, no significant interaction effect was observed (Table S1).

### 3.6 Age‐associated global proteomic differences and persistent immune activity at 28 days post‐injury

**FIGURE 6:**
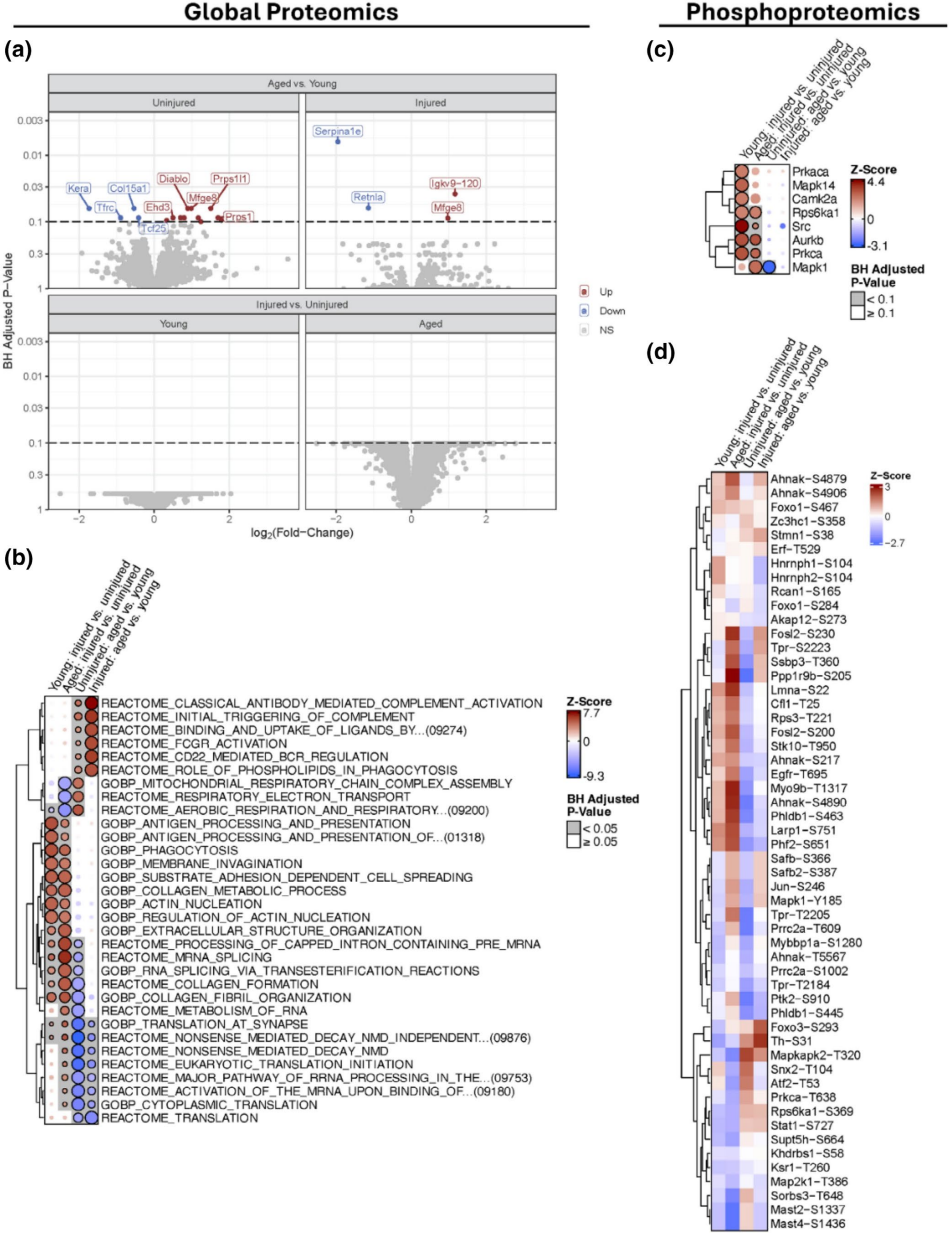
Age‐ and injury‐associated changes in global and phosphoproteomic profiles at day 28 post‐injury. (a) Volcano plots of differential proteins (*p* < 0.1) between ages within each injury status (top) and between injury statuses within each age group (bottom). Proteins enriched in muscles from young mice are shown in blue and those enriched in aged mice in red. (b, c) Bubble plots of enriched pathways from the Reactome and Gene Ontology Biological Processes databases (b), and enriched kinase‐associated phosphoprotein groups based on PhosphositePlus curations (c) across age and injury comparisons. Bubble color reflects *z*‐scores and bubble size corresponds to significance; gray shading indicates significant enrichment (Global: *p* < 0.05; Phospho: *p* < 0.1). (d) Heatmap of phosphproteins driving Mapk1 enrichment from Kinase Substrate Enrichment Analysis (KSEA). All analyses were performed using CAMERA‐PR and *p*‐values were adjusted across contrasts using the BH‐procedure.

Exploratory global and phosphoproteomic analyses were performed on injured (Young = 4, Aged = 5) and contralateral control (Young = 4, Aged = 5) TA muscles collected 28 days post‐injury to characterize age‐dependent molecular and signaling alterations during the chronic phase of VML recovery. At this late post‐injury stage, global protein profiles were largely stable (BH‐adjusted *p* > 0.1). However, several age‐associated differences in immune‐ and remodeling‐related proteins persisted (Table S2). In injured muscles from aged versus young mice, young mice displayed higher abundance of the serine protease inhibitor Serpina1e (3.9‐fold, *p* = 0.0062) and Retnla/FIZZ1 (2.2‐fold, *p* = 0.061), a marker of M2 macrophage activation and Th2‐driven remodeling (Sandler et al., 2003). Aged and injured muscles showed increased levels of the immunoglobulin Igkv9‐120 (2.3‐fold, *p* = 0.038) and milk fat globule epidermal growth factor (MFG‐E8, 2.0‐fold, *p* = 0.088) which is associated with phagocytosis and muscle wasting (Figure 6a, top right) (Guan et al., 2025; Ikemoto‐Uezumi et al., 2022). In uninjured muscles from aged versus young mice, MGF‐E8 remained significantly enriched (2.0‐fold, *p* = 0.062) alongside proteins related to nucleotide synthesis (Prps1, 3.2‐fold, *p* = 0.062) and apoptosis (Diablo, 1.9‐fold, *p* = 0.062). In uninjured young mice, proteins related to basement membrane structure and muscle quality (Col15a1, 1.5‐fold, *p* = 0.062), endocytic trafficking (Trfc, 1.9‐fold, *p* = 0.086), and ribosome quality control (Tcf25, 1.3‐fold, *p* = 0.086) were significantly more abundant (Figure 6a, top left). No proteins were significantly altered (*p* < 0.1) between injured and uninjured limbs within each age group (Figure 6a, bottom).

Building on this differential analysis, pathway‐level enrichment was performed using reference Reactome and Gene Ontology Biological Processes (GOBP) gene set curations. When comparing the injured muscle in aged versus young mice, enrichment of classical antibody mediated complement activation (*z* = 7.67, *p* = 1.19E‐11) and related immune pathways (e.g., Fc‐gamma receptor activation and CD22‐mediated B‐cell receptor regulation) was noted in aged animals and enrichment of translation and mRNA surveillance pathways was noted in young animals. In aged mice, injured muscles showed significant downregulation of several metabolic pathways relative to uninjured controls (e.g., mitochondrial respiratory chain complex assembly, *z* = −5.28, *p* = 0.00014), whereas no comparable suppression was detected in young mice. Across both ages, injury persistently enriched extracellular (e.g., extracellular structure organization, *z* = 3.91–5.23, *p* = 0.00014–0.000958), cytoskeletal (e.g., regulation of actin nucleation, *z* = 4.33–4.69, *p* = 0.0011–0.0027), and nuclear remodeling pathways, as well as immune surveillance and antigen handling (e.g., phagocytosis, *z* = 4.96–5.65, *p* = 0.00022–2.92E‐5), indicating persistent structural and immune activity even at 28 days post‐injury (Figure 6b and Table S3). Pathway enrichment analysis utilizing the CellMarker (Hu et al., 2023) database, which is curated to murine cellular populations, further confirmed sustained immune involvement following VML injury in both young and aged mice. Enrichment was particularly observed among macrophage and monocyte populations (*p* < 0.05, Figure S2A). The Pathway Interaction Database (Schaefer et al., 2009) also identified age‐ and injury‐associated differences in adhesion‐related pathways, including integrin β1/β3 signaling and adherens junction stability. Class II histone deacetylase‐associated signatures (HDACs), regulators of myogenic gene expression, showed contrasting patterns in the PID dataset, with reduced representation in uninjured muscles from aged versus young mice (*z* = −4.03, *p* = 0.0097) and increased representation in injured versus uninjured muscle from aged mice (*z* = 3.34, *p* = 0.022, Figure S2B and Table S3). Given the reported roles of HDACs in muscle aging and injury responses (Spallotta et al., 2013; Walsh & van Remmen, 2016), these patterns may reflect age‐dependent shifts in the regulation of regenerative transcriptional programs. These findings suggest that, despite minimal differential protein abundance 28 days post‐injury, age and injury continue to influence immune, metabolic, and structural processes relevant to longer‐term muscle recovery.

### 3.7 Phosphoproteomic signatures reveal altered stress‐responsive signaling in aged muscle

To further understand age‐dependent molecular responses during recovery from VML, phosphoproteomic profiling was performed. While no significant differences in individual phosphopeptides were observed between groups (Figure S3A), several sites including those on multiple actin isoforms and on calsequestrin‐1, a calcium‐handling protein, showed moderate fold changes that may warrant investigation in future, better‐powered studies (Figure S3B and Table S4). Although phosphosite level differences were modest, pathway‐level enrichment analysis using Kinase Substrate Enrichment Analysis (KSEA), which curates changes in phosphopeptides to their regulatory kinases (Wiredja et al., 2017), revealed differential enrichments between groups (Table S5).

Notably, differential kinase activity of Mapk1 was detected using both CAMERA‐PR and PTM‐SEA (Figure 6c and Figure S3C). Mapk1, a stress‐responsive kinase linked to remodeling and autophagy (Kong et al., 2019; Roux Philippe & Blenis, 2004), displayed contrasting phosphorylation patterns across conditions. In uninjured muscles, pathway activity was higher in young compared to aged mice (*z* = −3.02, *p* = 0.051), whereas in aged mice, injured muscles exhibited hyperactivation relative to uninjured (*z* = 2.72, *p* = 0.04, Figure 6c and Table S5).

To further resolve the features driving this signature, individual phosphoproteins contributing to the Mapk1‐associated node were examined. Several sites displayed potential biological relevance to muscle injury and aging. Cofilin‐1 T25, a residue whose phosphorylation can impair sarcomere structure and force generation, displayed a trend toward enrichment (*z* = 1.9, *p* = 0.17) in injured versus uninjured muscles in aged mice but was reduced in uninjured muscles from aged versus young mice (Vignier et al., 2021). This site is also notable given the association of cofilin with laminin (Lmna) perturbations, another differentially phosphorylated protein in this dataset. Fosl2 S230 (Fra2), a component of the AP‐1 transcription factor complex expressed in Pax7+ satellite cells, was likewise reduced in uninjured muscles in aged versus young mice, but disproportionately increased following injury in aged mice (*z* = 2.6, *p* = 0.14). Phosphosites near to S230 have been linked to Fosl2 protein stabilization, and its dysregulation may reflect age‐dependent alterations to satellite cell differentiative capacity (Alli et al., 2013). Ahnak phosphorylation (S217, S4879, S4890, S4906) also displayed enrichment in aged mice between injured and uninjured muscles. Ahnak is linked to p38α‐responsive signaling and may contribute to myogenic gene expression programs (Knight et al., 2012). Phosphorylation of Foxo3, another protein linked to muscle differentiation (Gellhaus et al., 2023), appeared to be more injury‐ than age‐dependent, though its specific phosphosite (S293) has not been investigated in relation to muscle (Figure 6d and Table S4). Together, these phosphoproteins reinforce Mapk1‐associated signaling as a potential node linking age‐ and injury‐dependent differences in muscle repair.

Additional injury‐associated kinase signatures of interest included Mapk14 and Prkaca, both of which were more enriched in young mice between injured and uninjured muscles than in the corresponding aged comparison (Figure 6c and Table S5). Feature‐level inspection of phosphoproteins contributing to these signatures highlighted several sites of biological interest. Mapk14 Y182, the canonical activation‐loop residue required for p38 Mapk activity, and Atf2 T53, a downstream p38‐responsive regulatory site, were both represented. Members of the p38 Mapk family play well‐established roles in skeletal muscle myogenesis and muscle function (Brennan et al., 2021; Segalés et al., 2016). Mapk14 Y182 phosphorylation was elevated in muscles from aged mice compared to young mice regardless of injury status (*z* = 2.2), but within each age group showed reduced phosphorylation following injury (*z* = −2.1 to −1.8) (Figure S3D and Table S4). Prkaca‐associated features included Plcl1 S96, a phosphosite that may warrant future investigation (Figure S3E). In inflammatory conditions such as rheumatoid arthritis, Prkaca activity has been linked to muscle remodeling, skeletal muscle mass maintenance, skeletal muscle contraction, insulin signaling, and platelet inactivation (Kim et al., 2025), suggesting potential relevance to the post‐injury regenerative environment. Collectively, these phosphoproteomic datasets reveal age‐dependent differences in age‐ and injury‐responsive signaling networks that were not evident from global protein abundance alone and identify stress‐responsive kinase activity as a key point of divergence.

## 4 DISCUSSION

To design tissue engineered therapies for volumetric muscle loss (VML) that are effective across the lifespan, it is essential to understand how aging mechanistically modifies an already dysregulated repair process. Although peak muscle force and morphometric outcomes appeared broadly similar across ages, suggesting that the severity of the VML injury may mask age‐related differences at the tissue level, molecular analyses revealed clear age‐associated alterations. The 40% VML defect was selected for this study to minimize spontaneous recovery and establish sustained functional deficits for future therapeutic testing, consistent with prior work using larger TA ablation models (Quarta et al., 2017; Vega‐Soto et al., 2020); however, a smaller or non‐critically sized defect may be useful in future studies to better identify age‐associated mechanisms that distinguish successful versus failed regeneration. Even with this severe injury model, aging was associated with distinct differences in contraction kinetics, inflammatory cytokine dynamics, and sustained shifts in complement signaling, metabolic pathways, and Mapk activity. These findings indicate that even when gross regenerative outcomes converge in the context of severe injury, aging modifies key molecular and cellular programs that are likely to determine the responsiveness of therapeutic interventions. Consistent with this, prior work suggests that the aged microenvironment may reduce the effectiveness of VML repair strategies such as regenerative rehabilitation (Habing et al., 2024). As such, successful translation may require combinatorial approaches that account for the aged context, for example by incorporating immunomodulatory components alongside existing tissue engineered therapies to create age‐optimized treatment strategies.

A key functional and translational metric in VML studies is peak muscle force. In this study, peak isometric force was similar across ages, a finding that may initially appear counterintuitive but is consistent with previous reports showing that size‐adjusted isometric strength can be preserved with aging. In humans, aging is more strongly associated with shifts in the force‐velocity relationship (reflecting reduced muscle shortening speed at a given load), declines in power output (force × velocity), and alterations in cross‐bridge kinetics (slower myosin attachment and detachment rates). Several studies have also reported that these kinetic impairments are more pronounced in women (Callahan & Kent‐Braun, 2011; Miller et al., 2013). In mice, age‐related reductions in motor unit connectivity can occur before or alongside measurable losses in strength (Sheth et al., 2018), suggesting that the manifestation of age effects depends on the extent of aging and on the specific functional readout assessed (e.g., grip strength versus plantar flexion isometric torque). Collectively, these studies support a model in which aging primarily affects the dynamics of muscle contraction, through changes in excitation‐contraction coupling and cross‐bridge cycling, rather than maximal isometric force generation alone, highlighting mechanisms that could be probed more directly in future work.

It is important to note that the literature on age‐associated changes in human muscle contractile kinetics is highly variable. Under ideal conditions, some studies report decreases in specific force (force normalized to muscle size) and in unloaded shortening velocity (maximum shortening speed under zero load) (D'Antona et al., 2003; Frontera et al., 2000; Ochala et al., 2007; Yu et al., 2007), whereas others observe minimal or no change (Frontera et al., 2008; Korhonen et al., 2006). In the present VML model, TA muscle relaxation rates were faster in aged mice, both with and without injury, which contrasts with the commonly reported slowing of relaxation with age and therefore warrants validation. Changes in the phosphorylation of proteins associated with calcium handling (e.g., Calsequestrin‐1) and structural regulation (e.g., Actins) may contribute to these altered relaxation dynamics, as might damage to the neuromotor junction. Given that impaired nerve regeneration is a major barrier to functional recovery following VML (Sorensen et al., 2021), future studies that include larger cohorts and additional time points should directly investigate neuromuscular junction remodeling and determine whether age‐dependent differences in denervation or reinnervation contribute to altered kinetics. Additional mechanisms, including calcium handling, cross‐bridge cycling, and fiber type composition, should also be investigated to clarify the basis of this observation. As peak force alone may not capture age‐ or injury‐related differences in muscle performance, subsequent assessments of regenerative and therapeutic outcomes should incorporate kinetic parameters alongside conventional strength measures to provide a more comprehensive view of functional recovery.

Additionally, interpretation of functional outcomes in this study should consider the use of the contralateral limb as a control rather than an uninjured animal. Although commonly used in VML studies, this approach represents a limitation particularly in the context of aging, as contralateral limbs can undergo compensatory adaptations, such as load‐induced hypertrophy, that may differ with age and complicate interpretation of normalized functional data. Contralateral controls were selected in this study to reduce animal number and cost and to minimize inter‐animal variability. Future studies incorporating independent uninjured controls will help distinguish true age‐related differences in muscle function following injury. Further investigation of compensatory versus natural limb loading may also hold translational relevance, as altered limb use after unilateral injury can reflect real‐world functional adaptations following trauma or surgery.

Collagen deposition presented another point of divergence from prior reports on age‐related injury responses. At 28 days post‐VML, Masson's Goldner staining showed the expected increases in collagen content relative to baseline, consistent with fibrotic rather than regenerative healing; however, collagen area did not differ by age. This finding is noteworthy given that aging is often associated with aberrant extracellular matrix accumulation (Alnaqeeb et al., 1984), and other VML models in rats have reported greater injury‐induced fibrosis in the muscles of aged mice, often in a collagen type‐specific manner (Kim et al., 2016, 2019).

Trichrome‐staining methods, while useful for quantifying total collagen, may not capture more subtle features such as isoform balance (type I versus III), collagen fiber maturity, and smaller scale regional heterogeneity, which could reduce sensitivity to age‐dependent differences in this study. Supporting the histological findings, pathway‐level proteomic analysis at day 28 post‐injury similarly showed injury‐driven, but age‐independent, enrichment of processes related to “collagen metabolism” and “extracellular matrix organization.” Notably, some ECM‐related signatures were age‐dependent with “collagen formation” pathways and Col15a1, a nonfibrillar basement membrane collagen important for microvascular and muscle fiber integrity (Eklund et al., 2001), both reduced in uninjured muscles from aged mice. These baseline differences suggest that aging alters ECM composition and organization in ways not reflected by bulk collagen staining.

Together, these findings suggest that aging alters collagen synthesis and matrix organization at the molecular level, even though bulk fibrotic remodeling appears similar across ages by day 28 post‐injury. Thus, age‐related effects may be reflected more in collagen quality, or the timing and regulation of early remodeling events rather than in total collagen accumulation at the study endpoint. Given the strong influence of fibrosis on muscle function (Dolan et al., 2022), and evidence for additional age‐ and injury‐associated alterations in the extracellular matrix, further work is warranted. Future studies should incorporate collagen type‐specific immunostaining, biochemical quantification of total collagen and cross‐linking, and earlier post‐injury time points to delineate the temporal progression and qualitative differences of fibrotic deposition with age.

A central driver of fibrosis in both VML and aging is dysregulation of the immune response. In this study, cytokine dynamics differed with both age and injury. IL‐6 is a canonical injury cytokine with dual pro‐inflammatory and reparative roles, and its amplitude and timing help recruit immune and muscle satellite cells to the site of injury (Muñoz‐Cánoves et al., 2013). In aged mice, chronic low‐grade inflammation was associated with a blunted post‐injury IL‐6 surge, potentially limiting effective IL‐6‐mediated cell recruitment and coordination of the regenerative response. IL‐6 can also activate Mapk signaling downstream of its receptor complex (Heinrich et al., 2003), suggesting that age‐related differences in IL‐6 dynamics may contribute to the shifts in Mapk1 activity observed in this study.

Equally important is the transition from a pro‐inflammatory to a pro‐reparative, anti‐inflammatory phase. Others have shown that this resolution phase is disrupted following VML, with persistent competing pro‐ and anti‐inflammatory cues contributing to chronic inflammation and fibrosis (Hymel et al., 2023). In this study, anti‐inflammatory mediators such as IL‐10 could not be reliably quantified, underscoring the need for future work that directly measures anti‐inflammatory signaling and profiles key immune cell populations, including neutrophils, macrophage subsets, and T cells, to better define how aging modulates immune resolution after VML.

Global proteome analysis reinforced the cytokine profiling results, revealing persistent enrichment of complement and phagocytic programs, with an age‐dependent difference in injury responses. Beyond inflammation, the complement system mediates opsonization, membrane attack complex formation, lysis, and phagocytic clearance (Janeway et al., 2001). These features have practical implications as transplanted cells may be cleared more rapidly in the muscles of aged mice, narrowing the therapeutic window, and beneficial paracrine signaling from transplanted cells may be attenuated by the background inflammatory milieu. Accordingly, future aged VML studies should consider evaluating myogenic therapies in combination with immune modulation and include earlier time points for global proteomic profiling to further capture transient differences in immune and myogenic programs.

Phosphoproteomic analyses point to Mapk1 as a stress‐responsive signaling node that differs with age and VML injury. Several features of the Mapk1‐associated signature align with myogenic regulatory programs. For example, hyper‐phosphorylation of proteins including Fosl2 and Ahnk in aged mice following injury suggests that, while individual phosphosite changes were modest, their collective pattern reflects age‐dependent alterations in stress‐responsive pathways engaged during muscle repair (Alli et al., 2013; Knight et al., 2012). The persistence of these phosphorylation patterns at this late post‐injury time point further suggests that muscles from aged mice may maintain these stress‐related signaling programs longer than young mice, potentially reflecting delayed resolution of the repair process. Additionally, trends in cofilin‐1 phosphorylation warrant future investigation, as cofilin‐mediated remodeling may contribute to the age‐ and injury‐related differences in contractile dynamics observed in this study (Vignier et al., 2021).

From a therapeutic perspective, the identification of age‐dependent Mapk1‐associated phosphorylation may inform the design of combinatorial regenerative rehabilitation strategies. Resistance exercise is well characterized as an intervention to preserve muscle mass and function with aging (Hurst et al., 2022) and can increase phosphorylation of Mapk pathway components, including Erk1/2, p90rsk, Jnk, and p38‐Mapk (Lee & Nicoll, 2023). Therefore, regenerative strategies that incorporate resistance‐based loading may provide a means to modulate stress‐responsive signaling pathways that are altered in aged muscle following VML. However, because Mapk signaling can contribute to both adaptive remodeling and prolonged stress responses, future work is needed to determine whether exercise‐induced modulation of these pathways improves regeneration and how it may be best integrated with scaffold‐, cell‐, or drug‐based repair strategies.

In addition to rehabilitation‐based modulation of Mapk signaling, upstream mediators of Mapk pathways may provide more direct molecular targets for adjuvant therapies. MFG‐E8, which was elevated with age in both injured and uninjured muscles and is a known activator of Erk/Mapk (Guan et al., 2025), represents one such candidate. Comprehensively characterized as component of arterial aging, MFG‐E8 promotes pro‐inflammatory phenotypic shifts and invasive behavior in vascular smooth muscle cells, linking it to adverse vascular remodeling (Ni et al., 2022). In skeletal muscle, the effects of MFG‐E8 are less clearly defined. In vitro studies suggest pro‐myogenic effects through PI3K/Akt and Erk signaling (Li et al., 2017; Li et al., 2019), and in aging rat models administration of MFG‐E8 was associated with enhanced mitochondrial biogenesis, reduced lipid accumulation, and increases in muscle size (Guan et al., 2025; Li et al., 2024). Conversely, MFG‐E8 accumulates at neuromuscular junctions (NMJ) in aged muscles prior to denervation, and knockdown was reported to lessen age‐related NMJ degeneration and muscle loss (Ikemoto‐Uezumi et al., 2022). Alongside an unresolved, possibly compensatory role in myogenesis, MFG‐E8 is linked to phagocytotic programs via its role in efferocytosis (engulfment and digestion of apoptotic cells) (Szondy et al., 2022). Thus, by coupling immune clearance, Erk/Mapk signaling, and regeneration, MFG‐E8 is a plausible context dependent regulator of age‐related repair and a tractable translational target for testing in future aged VML models. Prior work demonstrates the feasibility of systemic MFG‐E8 administration (e.g., orally) (Guan et al., 2025), although these studies focused on sarcopenia treatments rather than regeneration following injury, and further work is needed to define its role in repair. As a secreted protein within skeletal muscle (Chikazawa et al., 2020), MFG‐E8 may also be compatible with localized biomaterial‐based delivery to modulate the post‐injury microenvironment and regenerative activity. Prior multi‐omic VML studies have also identified SP1 as a potential regulatory node (Jain et al., 2025). SP1 activity can be mediated by the Mapk pathway (Milanini‐Mongiat et al., 2002; Tan & Khachigian, 2009), and its protein abundance decreases with age (Swift et al., 2022), making it another compelling target for follow‐up in aged VML studies.

It is important to note that these proteomic and phosphoproteomic datasets were generated from a pilot‐sized cohort and collected at a relatively late stage of healing (28 days post‐injury) without baseline comparison, factors that likely reduced the statistical power to detect individual protein‐levels differences in abundance and phosphorylation. Many injury‐dependent responses occur earlier in the regenerative timeline and may not be captured on day 28. Accordingly, future studies incorporating larger cohorts and earlier time points will be essential for resolving transient signaling pathways that differ between young and aged injured mice. Even so, the integrated multi‐omic patterns identified herein reveal persistent age‐associated alterations in stress‐responsive and immune signaling pathways, underscoring the value of this dataset for generating mechanistic hypotheses and guiding the design of future studies aimed at improving muscle repair in aged populations. Future studies of temporal multi‐omics changes in young and aged muscle following VML, alongside therapy‐induced molecular remodeling (e.g. cellular and scaffold therapies and exercise), will help identify therapeutic targets to enhance regeneration in aged muscle.

## 5 CONCLUSION

Following volumetric muscle loss, aging is associated with persistent differences in immune programs and Mapk signaling that remain evident even when overt functional and morphometric deficits appear comparable. These findings emphasize that aging reshapes the underlying repair milieu, and future therapeutic strategies should account for age‐dependent molecular contexts that may influence regenerative outcomes.

## AUTHOR CONTRIBUTIONS

**Krista M. Habing:** Conceptualization; data curation; formal analysis; investigation; methodology; validation; visualization. **Tyler J. Sagendorf:** Conceptualization; data curation; formal analysis; methodology; validation; visualization. **Cynthia A. Alcazar‐Daleo:** Conceptualization; data curation; formal analysis; methodology. **James A. Sanford:** Data curation; formal analysis; methodology. **Damon Leach:** Data curation; formal analysis; methodology. **Joshua C. Vanderpool:** Data curation; formal analysis; methodology. **Chelsea M. Hutchinson‐Bunch:** Data curation; formal analysis; methodology. **Marina Gritsenko:** Data curation; formal analysis; methodology. **Gina M. Many:** Conceptualization; data curation; formal analysis; funding acquisition; methodology; resources; supervision; validation; visualization. **Karina H. Nakayama:** Conceptualization; funding acquisition; methodology; resources; supervision; visualization.

## FUNDING INFORMATION

This work was supported in part by the National Institutes of Health/National Institute of Arthritis and Musculoskeletal and Skin Diseases under Grant No. R01AR080150 (to K.H.N.), National Institutes of Health/National Center for Advancing Translational Sciences under Grant No. TL1TR002371 (to K.M.H.), and MTF Biologics (to K.H.N.). Additionally, this work was partially supported through a Pacific Northwest Biomedical Innovation Co‐Laboratory (PMedIC) project funded by the Laboratory Directed Research and Development Program at Pacific Northwest National Laboratory, a multiprogram national laboratory operated by Battelle for the Department of Energy (to G.M.M. and K.H.N.).

## CONFLICT OF INTEREST STATEMENT

No conflicts of interest, financial or otherwise, are declared by the authors.

## ETHICS STATEMENT

All animal procedures were approved by the Oregon Health & Science University Institutional Animal Care and Use Committee (Protocol Number: TR01_IP00002839).

## Supporting information


**Figure S1:** Differential frequency dependence of muscle force and contraction kinetics. (a–c) Frequency sweeps of normalized peak muscle force (a), contraction rate (b), and relaxation rate (c) of injured (darker color) and uninjured (lighter color) tibialis anterior muscles in young (blue) and aged (green) mice. Shown are mean ± SD.

**Figure S2:** Additional pathway enrichment heatmaps from age and injury comparisons. (a, b) Top CellMarker cell‐type (a) and Pathway Interaction Database (PID) signatures (b) significantly enriched (*p* < 0.05) in any of the four comparisons. Bubble color indicates *z*‐score and bubble size corresponds to significance, with more significant terms represented by larger bubbles. A gray background denotes significance (Young = 4, Aged = 5, both TA muscles). Significance was determined using CAMERA‐PR and *p*‐values were adjusted using the BH procedure.

**Figure S3:** Additional phosphoproteomic analyses. (a) Volcano plots of significantly enriched (*p* < 0.1) phosphosites across age and injury conditions. (b) Table of phosphoproteins of interest with a BH‐adjusted *p*‐value of approximately 0.11 and a log fold change greater than 2. (c) Enriched (*p* < 0.1) phosphosite signatures identified using Post‐Translational Modification Signature Enrichment Analysis (PTM‐SEA). Bubble color represents the normalized enrichment score (NES) and bubble size is scaled by significance. A gray background denotes significance (Young = 4, Aged = 5, both TA muscles). (d, e) Heatmaps showing the top phosphoproteins and their corresponding phosphorylation sites within the Mapk14 (c) and Prkaca (d) kinase‐associated groups highlighted by CAMERA‐PR enrichment.

**Table S1:** Summary of Two‐Way ANOVAs.

**Table S2:** Global proteome dataset used for differential abundance analysis.

**Table S3:** Phosphoproteome dataset used for phosphosite‐level differential analysis.

**Table S4:** Global CAMERA pathway enrichment analysis.

**Table S5:** Phosphoproteomic CAMERA pathway enrichment analysis.

**Video S1:** Muscle force contraction of an uninjured tibialis anterior muscle during in situ muscle physiology.

**Video S2:** Muscle force contraction of an injured tibialis anterior muscle during in situ muscle physiology.

## Data Availability

The data that support the findings of this study will be available from the corresponding author upon reasonable request. Proteomics data have been deposited in the MassIVE repository under accession number MSV000102175.
