# Supplementary material for: Cellular signaling within aged skeletal muscle reveals a dysregulated stress‐induced remodeling response following volumetric muscle loss in female mice
Source: Physiol Rep. 2026 Jul 23;14(14):e71022. doi: 10.14814/phy2.71022 (PMC13396886; doi:10.14814/phy2.71022)
Supplement: Supplementary file 1 — Figure S1: Differential frequency dependence of muscle force and contraction kinetics. (a–c) Frequency sweeps of normalized peak muscle force (a), contraction rate (b), and relaxation rate (c) of injured (darker color) and uninjured (lighter color) tibialis anterior muscles in young (blue) and aged (green) mice. Shown are mean ± SD. [file PHY2-14-e71022-s010.docx]

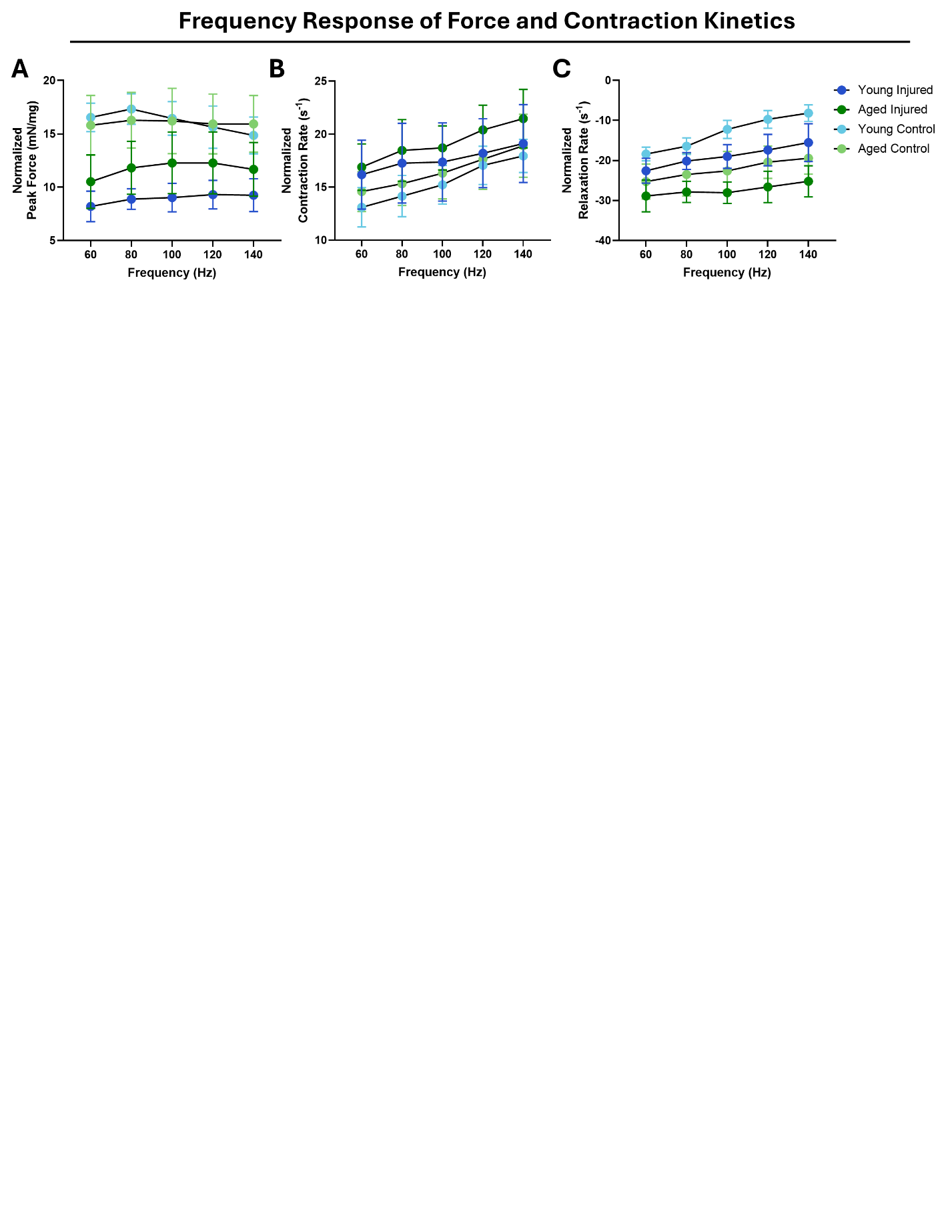


**Supplemental Figure S1. Differential frequency dependence of muscle force and contraction kinetics. A-C)** Frequency sweeps of normalized peak muscle force (**A**), contraction rate (**B**), and relaxation rate (**C**) of injured (darker color) and uninjured (lighter color) tibialis anterior muscles in young (blue) and aged (green) mice. Shown are mean ± SD.
