# Supplementary material for: Cellular signaling within aged skeletal muscle reveals a dysregulated stress‐induced remodeling response following volumetric muscle loss in female mice
Source: Physiol Rep. 2026 Jul 23;14(14):e71022. doi: 10.14814/phy2.71022 (PMC13396886; doi:10.14814/phy2.71022)
Supplement: Supplementary file 2 — Figure S2: Additional pathway enrichment heatmaps from age and injury comparisons. (a, b) Top CellMarker cell‐type (a) and Pathway Interaction Database (PID) signatures (b) significantly enriched (p < 0.05) in any of the four comparisons. Bubble color indicates z‐score and bubble size corresponds to significance, with more significant terms represented by larger bubbles. A gray background denotes significance (Young = 4, Aged = 5, both TA muscles). Significance was determined using CAMERA‐PR and p‐values were adjusted using the BH procedure. [file PHY2-14-e71022-s009.docx]

**
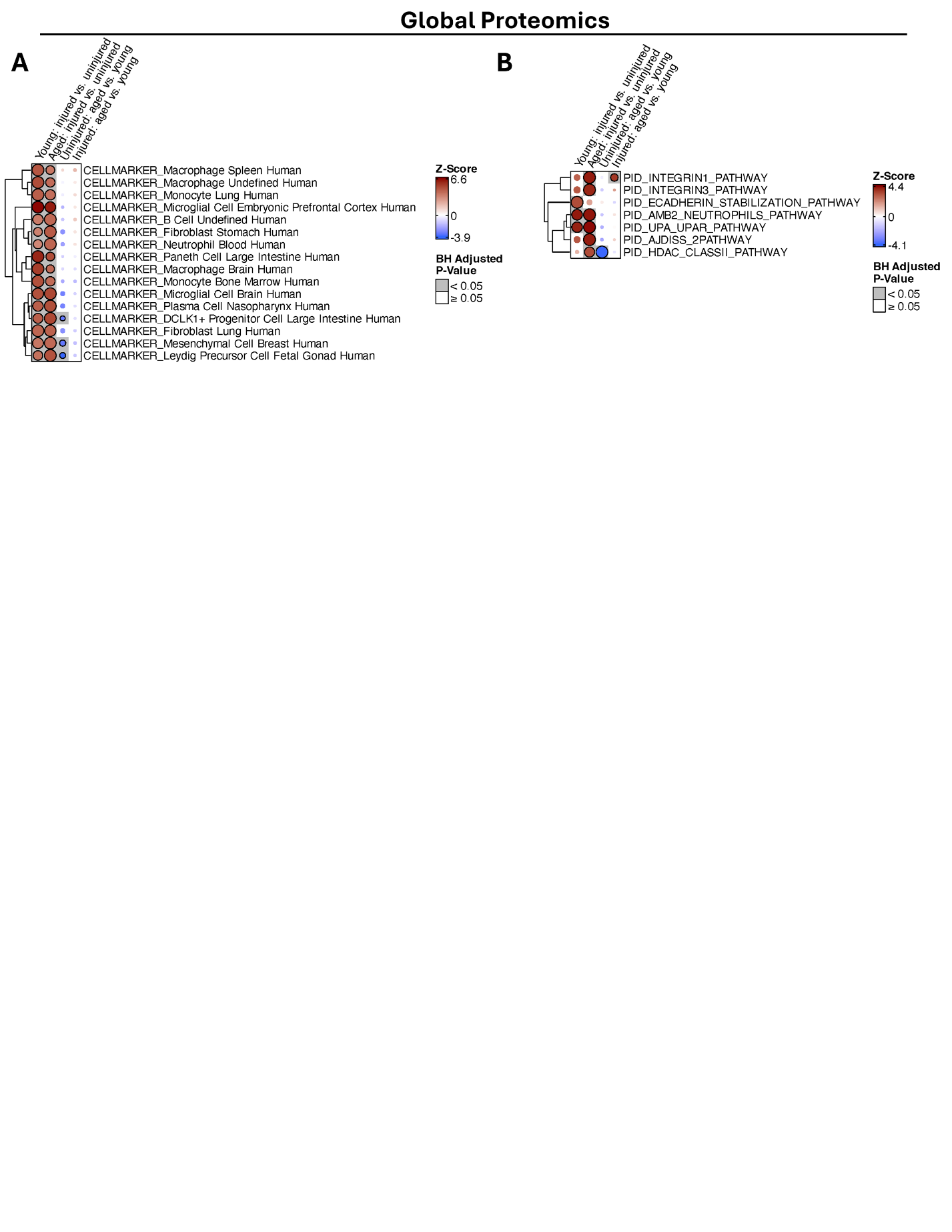
**

**Supplemental Figure S2. Additional pathway enrichment heatmaps from age and injury comparisons. A-B)** Top CellMarker cell-type (**A**) and Pathway Interaction Database (PID) signatures (**B**) significantly enriched (p < 0.05) in any of the four comparisons. Bubble color indicates z-score and bubble size corresponds to significance, with more significant terms represented by larger bubbles. A gray background denotes significance (Young = 4, Aged = 5, both TA muscles). Significance was determined using CAMERA-PR and p-values were adjusted using the BH procedure.
