# Supplementary material for: Cellular signaling within aged skeletal muscle reveals a dysregulated stress‐induced remodeling response following volumetric muscle loss in female mice
Source: Physiol Rep. 2026 Jul 23;14(14):e71022. doi: 10.14814/phy2.71022 (PMC13396886; doi:10.14814/phy2.71022)
Supplement: Supplementary file 3 — Figure S3: Additional phosphoproteomic analyses. (a) Volcano plots of significantly enriched (p < 0.1) phosphosites across age and injury conditions. (b) Table of phosphoproteins of interest with a BH‐adjusted p‐value of approximately 0.11 and a log fold change greater than 2. (c) Enriched (p < 0.1) phosphosite signatures identified using Post‐Translational Modification Signature Enrichment Analysis (PTM‐SEA). Bubble color represents the normalized enrichment score (NES) and bubble size is scaled by significance. A gray background denotes significance (Young = 4, Aged = 5, both TA muscles). (d, e) Heatmaps showing the top phosphoproteins and their corresponding phosphorylation sites within the Mapk14 (c) and Prkaca (d) kinase‐associated groups highlighted by CAMERA‐PR enrichment. [file PHY2-14-e71022-s008.docx]

**
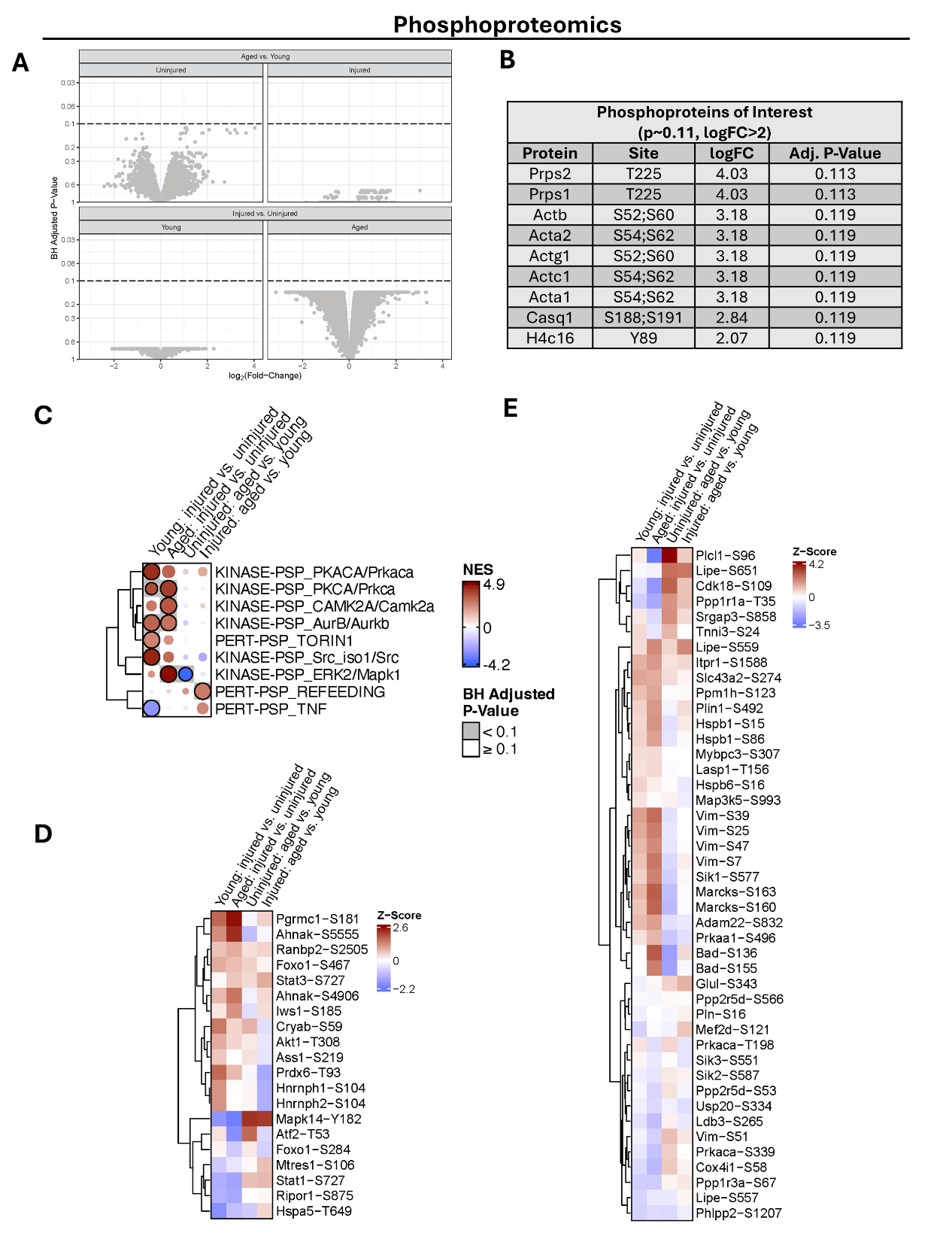
**

**Supplemental Figure S3. Additional phosphoproteomic analyses. A)** Volcano plots of significantly enriched (p < 0.1) phosphosites across age and injury conditions. **B**) Table of phosphoproteins of interest with a BH-adjusted p-value of approximately 0.11 and a log fold change greater than 2. **C)** Enriched (p<0.1) phosphosite signatures identified using Post-Translational Modification Signature Enrichment Analysis (PTM-SEA). Bubble color represents the normalized enrichment score (NES) and bubble size is scaled by significance. A gray background denotes significance (Young = 4, Aged = 5, both TA muscles). **D-E)** Heatmaps showing the top phosphoproteins and their corresponding phosphorylation sites within the Mapk14 (**C**) and Prkaca (**D**) kinase-associated groups highlighted by CAMERA-PR enrichment.
