# Supplementary material for: Cellular signaling within aged skeletal muscle reveals a dysregulated stress‐induced remodeling response following volumetric muscle loss in female mice
Source: Physiol Rep. 2026 Jul 23;14(14):e71022. doi: 10.14814/phy2.71022 (PMC13396886; doi:10.14814/phy2.71022)
Supplement: Supplementary file 4 — Table S1: Summary of Two‐Way ANOVAs. [file PHY2-14-e71022-s001.docx]

**Supplemental Table S1. Summary of Two-Way ANOVAs.** For terminal measures (histology and function), “injury” represents within-animal comparisons of injured versus contralateral limbs. For longitudinal measures (cytokine profiles and load bearing), “time” represents the temporal response to injury from baseline onwards.

|  | Main Effect: Age | Main Effect: Injury | Main Effect: Time | Interaction Effect |
| --- | --- | --- | --- | --- |
| **Cytokine Profiles** | | | | |
| G-CSF | Y |  | Y | N |
| Eotaxin | Y |  | Y | Y |
| IL-6 | Y |  | Y | Y |
| IP-10/CXCL10 | Y |  | Y | Y |
| KC/CXCL1 | Y |  | Y | N |
| MIG/CXCL9 | Y |  | N | Y |
| **Histology** | | | | |
| Muscle Area | N | Y |  | N |
| Collagen Area | N | Y |  | N |
| **Load Bearing** | | | | |
| Mouse Mass | Y |  | Y | Y |
| Time Mobile | Y |  | Y | Y |
| Rear Left % Body Weight | Y |  | Y | Y |
| Rear Left Pressure | Y |  | Y | N |
| Rear Left Time No Contact | N |  | Y | Y |
| Front/Rear Weight Ratio | Y |  | N | Y |
| Rear Left/Right Weight Ratio | Y |  | N | N |
| **Function** | | | | |
| Muscle Mass | N | Y |  | N |
| Peak Force | N | Y |  | N |
| Contraction Rate | N | Y |  | N |
| Relaxation Rate | Y | Y |  | N |
| Rate Ratio | Y | N |  | N |
| Contraction Duration | Y | Y |  | N |
